# A Geographic Mosaic of Climate Change Impacts on Terrestrial Vegetation: Which Areas Are Most at Risk?

**DOI:** 10.1371/journal.pone.0130629

**Published:** 2015-06-26

**Authors:** David D. Ackerly, William K. Cornwell, Stuart B. Weiss, Lorraine E. Flint, Alan L. Flint

**Affiliations:** 1 Department of Integrative Biology, University of California, Berkeley, California, United States of America; 2 Jepson Herbarium, University of California, Berkeley, California, United States of America; 3 School of Biological, Earth and Environmental Sciences, University of New South Wales, Sydney, Australia; 4 Creekside Center for Earth Observation, Menlo Park, California, United States of America; 5 Water Resources Division, United States Geological Survey, Sacramento, California, United States of America; University of New England, AUSTRALIA

## Abstract

Changes in climate projected for the 21^st^ century are expected to trigger widespread and pervasive biotic impacts. Forecasting these changes and their implications for ecosystem services is a major research goal. Much of the research on biotic responses to climate change has focused on either projected shifts in individual species distributions or broad-scale changes in biome distributions. Here, we introduce a novel application of multinomial logistic regression as a powerful approach to model vegetation distributions and potential responses to 21^st^ century climate change. We modeled the distribution of 22 major vegetation types, most defined by a single dominant woody species, across the San Francisco Bay Area. Predictor variables included climate and topographic variables. The novel aspect of our model is the output: a vector of relative probabilities for each vegetation type in each location within the study domain. The model was then projected for 54 future climate scenarios, spanning a representative range of temperature and precipitation projections from the CMIP3 and CMIP5 ensembles. We found that sensitivity of vegetation to climate change is highly heterogeneous across the region. Surprisingly, sensitivity to climate change is higher closer to the coast, on lower insolation, north-facing slopes and in areas of higher precipitation. While such sites may provide refugia for mesic and cool-adapted vegetation in the face of a warming climate, the model suggests they will still be highly dynamic and relatively sensitive to climate-driven vegetation transitions. The greater sensitivity of moist and low insolation sites is an unexpected outcome that challenges views on the location and stability of climate refugia. Projections provide a foundation for conservation planning and land management, and highlight the need for a greater understanding of the mechanisms and time scales of potential climate-driven vegetation transitions.

## Introduction

During periods of major climate change in the past, changes in the dominant vegetation were widespread and profound. The paleoecological record shows that from the Last Glacial Maximum to the pre-industrial period, global mean temperature rise of 3–8°C [1] has been accompanied by continental-scale shifts in species distributions. At the local scale these shifts were manifest as dramatic changes in the structure and function of plant communities (e.g., shifts from coniferous to broadleaf forest, forest to shrubland, etc.). Since the beginning of the industrial period (1850), global temperatures have risen almost 1°C, and climate models project additional increases of at least 1°C and up to 4°C or more, depending on the future trajectory of greenhouse gas emissions, land use change, and feedbacks in the global climate system [1]. The magnitude of projected 21^st^ century change is comparable to the warming since the end of the last ice age, and the rate of change is at least 10 times faster [2,3], suggesting that impacts on natural systems will be dramatic. Changes in the physiognomy and composition of dominant plant communities have profound effects on biodiversity, ecosystem function, conservation, and human welfare from local to global scales [4,5]. Understanding and forecasting these changes presents one of the more important challenges in ecology. In this paper, we address three current problems that arise in efforts to project vegetation responses to climate change at regional scales: 1) modeling the regional distribution of major vegetation types (rather than distributions of individual species), 2) assessing responses across a wide range of possible future climates; and 3) separating the contributions of variation in the magnitude (i.e. exposure) of climate change vs. variation in the sensitivity of different communities or species to the overall impacts.

Regarding the first point, a wide range of statistical and mechanistic approaches have been developed to model the distributions of individual species, communities and biomes from local to global scales (see reviews in [6,7]). The selection of appropriate methods depends on the source, quantity and nature of available data, as well as the objectives of the study. Species distribution models, focused on individual taxa, are widely used to address potential impacts of climate change, and inform conservation strategies. To complement these approaches, there is a need for improved methods that address the arrangement and cover of alternative habitats, biomes or vegetation types across a landscape or region [8]. Dynamic global vegetation models (DGVMs) provide one approach to vegetation modeling, incorporating varying degrees of physiological and ecological mechanisms [9,10]; the tradeoff is that DGVMs generally focus on a limited number of functional types (e.g., ‘deciduous broadleaf tree’), making it difficult to link the outputs to biodiversity mapping and conservation planning. Vegetation maps, based on the distribution of major vegetation types, play a complementary role as the types are defined based on dominant species, and reflect major physiognomic and functional characteristics of the associated ecosystems. These maps are becoming increasingly accurate and detailed with advances in remote sensing [11]. While vegetation types may be seen as artificial constructs in the context of community ecology, they play a central role in place-based land use and conservation planning [12]. Land managers are widely concerned with ‘type conversion’, shifts in dominant vegetation types due to disturbance (e.g., fire), succession or anthropogenic impacts, and their consequences for biodiversity conservation and resource management [13]. Factors such as disturbance history, priority effects, and vegetation-environment feedbacks can also lead to alternative vegetation types occurring under similar environmental conditions [14,15]. In this paper, we employ multinomial logistic regression to develop a probabilistic vegetation model (PVM) of the distribution of vegetation types at a regional scale under current and future climates, and explore several valuable features of this framework to address fundamental ecological questions with applications to regional conservation planning.

A second major challenge that arises in projections of biotic responses to climate is the uncertainty regarding the future trajectory of climate change in the 21^st^ century and beyond. Much of this uncertainty reflects socio-economic uncertainty regarding trajectories of population growth, land use change, and especially the rate and total amount of greenhouse gas emissions to the atmosphere [1]. In addition, global climate models vary in their sensitivity to greenhouse gases and treatment of critical feedbacks in the climate system. While projections of temperature rise are fairly consistent, models vary in projections of the amount and distribution of rainfall, especially at mid-latitudes such as California [16,17]. In the face of uncertainty, many studies either focus on a small number of future scenarios, such that each one can be examined and discussed individually (e.g., [18] and many others), or seek consensus or ensemble outputs across multiple scenarios [19]. We explore an alternative approach of considering a large number of future scenarios to assess the sensitivity of biotic responses to different climate scenarios (defined below), avoiding the need to select particular futures or approaches based on aggregate performance across an ensemble of models [20].

Lastly, the potential impacts of climate change on biological communities can be viewed as a function of the magnitude of climate change (exposure), the sensitivity of the organisms involved to a particular amount of change, and the adaptive capacity of a system to respond and offset potential impacts. This framework is widely used in social sciences [21,22] and is increasingly being applied to ecosystems and organisms [23]. At a landscape scale, a key ecological question with important conservation implications is how these components of climate impacts (exposure, sensitivity, and adaptive capacity) vary as a function of physical landscape factors (topography and hydrology), the location of individual populations (e.g., trailing vs. leading edge), or the ecology of different species and community types. Studies of climate refugia generally focus on landscape positions that are cool and/or moist (e.g., north-facing slopes) or on sites that may be more climatically or hydrologically stable (e.g., near water bodies or on deep soils) [24–27]. These locations may experience less exposure to climate change. However, even if cool and moist locations do support taxa that are threatened by a warming climate, that does not in itself indicate that these sites will be more stable or experience less biotic turnover. We use our probabilistic modeling framework together with the analysis of large numbers of future climate scenarios to develop a novel approach to assess magnitude and sensitivity of change in projected vegetation distributions, and partition the explanatory factors that influence the degree of sensitivity of vegetation across the landscape. The latter component allows us to test the hypothesis that cool and/or moist sites will experience less severe biotic impacts of climate change.

**Fig 1:**
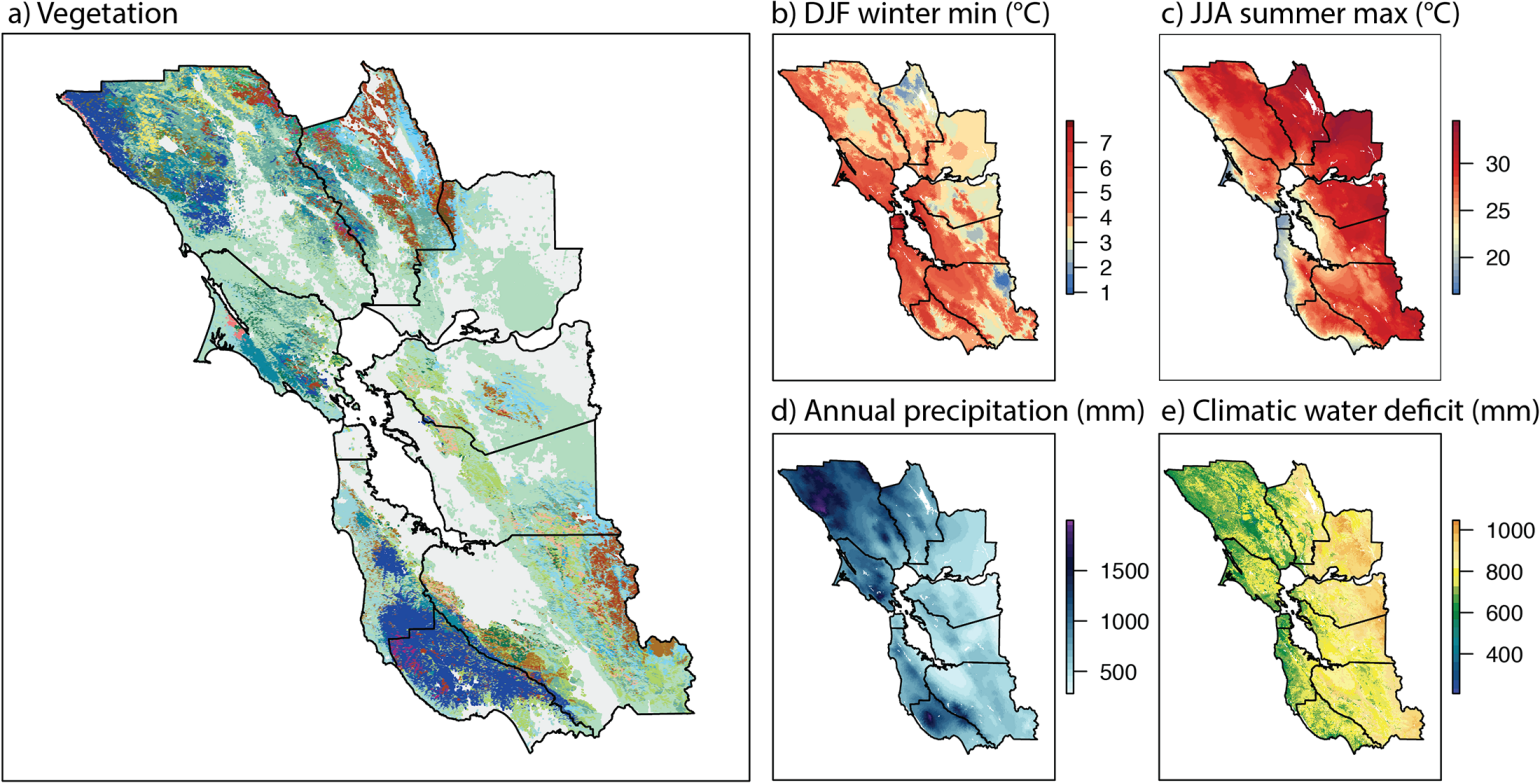
San Francisco Bay Area vegetation and climate. Maps of vegetation types, and the four climatic variables used as predictors for modeling the distribution of vegetation (maps of other predictors are shown in S1 Fig). A) Vegetation (see legend at top of Fig 3); gray areas on the map are urban and agricultural, or rare vegetation types not included in model. B-E) Climate variables, showing 1951–1980 historic norms. DJF = December, January, February. JJA = June, July, August. Borders delineate ten counties of San Francisco Bay Area.

Our study system is the vegetation of the San Francisco Bay Area (SFBA), California, USA (Fig 1A). The region covers almost 20,000 km^2^, with complex climate gradients, rugged topography, and exceptional biodiversity. It is situated in the center of the California Floristic Province, characterized by mediterranean-type climate with a strong maritime influence due to the proximity to the Pacific Ocean. The SFBA houses about 3000 native plant species, with 50 or so endemic to the region [28]. As part of a systematic conservation planning study, a high resolution vegetation map was recently developed for the region distinguishing several dozen different types of natural vegetation, as well as areas converted to urban, agricultural and other uses [29]. This map, together with recently developed high resolution spatial interpolations of climate and water balance variables (Fig 1B–1E) [30,31], provide the input data for our modeling effort, and the basis for projections of future vegetation distributions in response to climate change. In this study, we combine the methods and approaches outlined above to address the following questions:

1. What are the relative contributions of climatic, edaphic, and topographic factors, and an integrated assessment of climatic water deficit (sensu [32]), as predictors of contemporary vegetation distributions in the SFBA?
2. What are the magnitudes and patterns of projected climate change impacts on vegetation and vegetation change across our study region?
3. What are the relative contributions of changes in temperature (and associated impacts on water deficits) vs. precipitation across a wide range of future scenarios to the overall magnitude of projected change and to changes in the amount and distribution of individual vegetation types?
4. How do exposure to climate change and sensitivity of vegetation vary across the landscape, and what factors explain spatial variation in sensitivity to change? Specifically, we test the hypothesis that climate refugia (moist, north-facing slopes, cool coastal areas, or valleys with deep soils) will buffer responses to climate change and exhibit less impact in terms of projected vegetation change.

## Results

### Climate change

**Fig 2:**
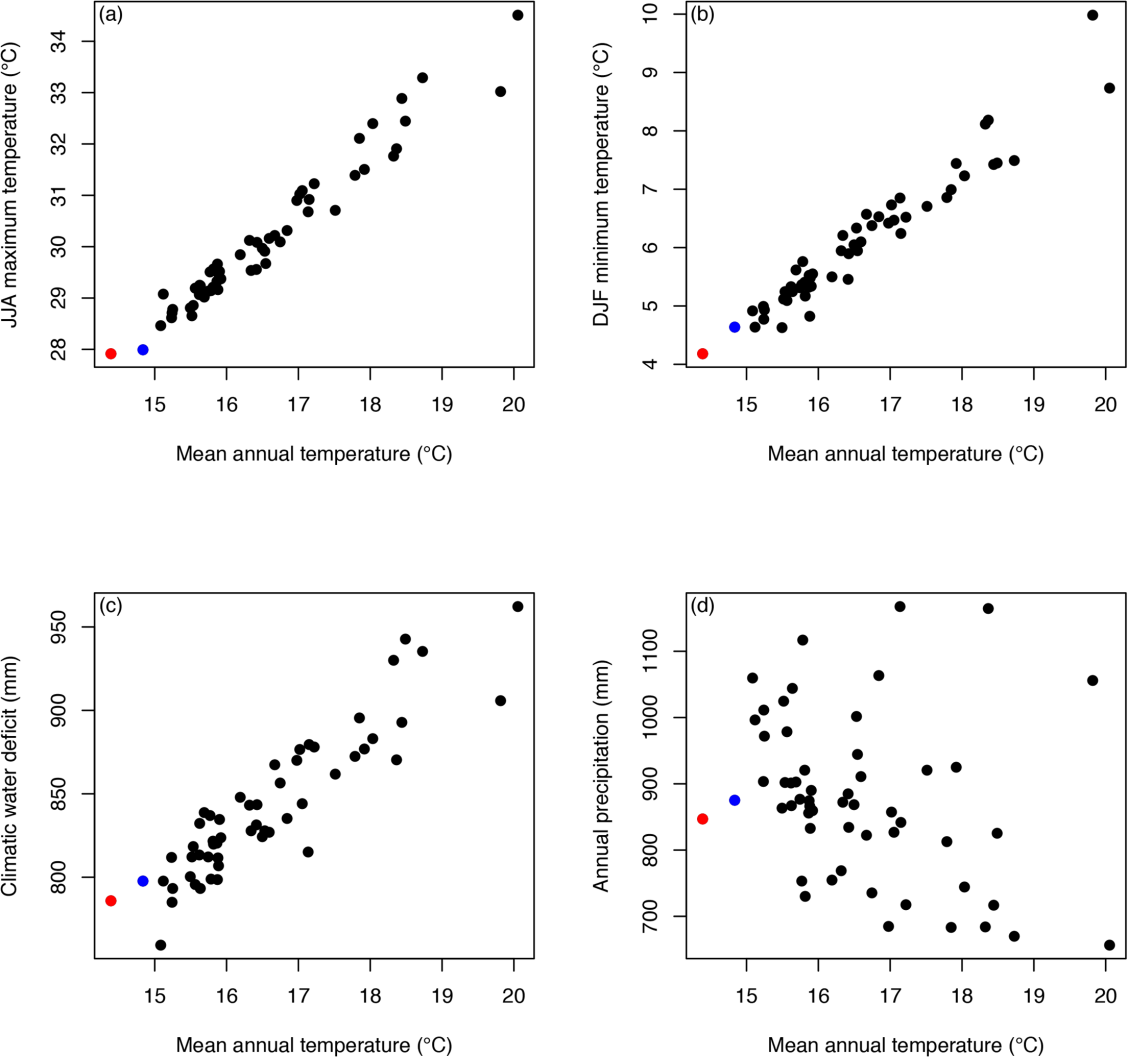
Historical and projected climate means. Thirty year climatic means vs. mean annual temperature for the historic (1951–1980, red) and recent (1981–2010, blue) periods and 54 possible futures (black) based on 18 different model/forcing scenarios and three time periods (2010–2039, 2040–2069, 2070–2099). See values in S3 Table.

Across the 54 climate futures examined here, mean summer maximum temperatures (June, July, August: JJA) increase up to 6.6°C, winter minimum temperatures (December, January, February: DJF) increase up to 5.8°C, climatic water deficit (CWD) increases up to 176 mm (+22.4%), and precipitation (PPT) varies from –23 to +38% (all values relative to 1951–1980 historical baselines, and based on spatial averages across 1 million sample points in our spatial domain; see Methods). For analysis and visualization, we rank models by their increase in mean annual temperature (MAT), though MAT was not used in the vegetation model. Increases in JJA, DJF, and CWD were all strongly correlated with MAT, while changes in PPT were uncorrelated with MAT (Fig 2).

### Model fit

Multinomial logistic regression, with 22 vegetation types (Table 1) and 7 predictors, correctly predicted the observed vegetation type in 52.0% of pixels (V_max_ = V_obs_) (Table 2, based on the training data set, N = 200,000). The importance of each of the seven predictors in the model was assessed by comparing ∆BIC values of the full model with reduced models, removing one term at a time. All factors made very strong contributions to the full model (∆BIC values > 10,000). The most important predictors were PPT and JJA and the least important factors were DJF and March radiation (Table 2). Removing factors had small effects on the percent of pixels correctly predicted, which was above 48% in all cases. Models with the four climate factors alone and the three fixed factors alone performed substantially worse (Table 2).

**Table 1:** Vegetation types.

Physiognomic types	Vegetation type (1G)	% area^1^
Grassland	Grassland (11)	35.9^2^
Shrubland	Chamise Chaparral (7)	3.24
	Mixed Chaparral (14)	0.52
	Mixed Montane Chaparral (15)	5.30
	Coastal Scrub (8)	3.58
	Semi-Desert Scrub (20)	1.59
Deciduous Woodland	Valley Oak Forest / Woodland (22)	0.24
	Blue Oak Forest / Woodland (4)	6.62
	Blue Oak-Foothill Pine Woodland (3)	1.12
	Oregon Oak Woodland (17)	1.31
	Black Oak Forest / Woodland (2)	0.14
Evergreen Woodland	Coast Live Oak Forest / Woodland (9)	8.27
	Interior Live Oak Forest/Woodland (12)	0.31
	Canyon Live Oak Forest (6)	0.25
	California Bay Forest (5)	1.69
	Tanoak Forest (21)	0.97
	Montane Hardwoods (16)	11.3
Conifer	Douglas Fir Forest (10)	5.89
	Bishop Pine Forest (1)	0.25
	Knobcone Pine Forest (13)	0.45
	Ponderosa Pine Forest (18)	0.40
	Redwood Forest (19)	10.6

List of 22 vegetation types included in this study under the ‘1G’ model, assignment to physiognomic classes, and percent of the study region covered by each type (based on [29]). Numbers after each vegetation type indicate alphabetical sort order, used in S3 Fig See list of dominant taxa in each vegetation type in S1 Table.

^1^Collectively, these occupy 61% of the terrestrial area in San Francisco Bay Area.

^2^Cool = 2.68%; Moderate = 5.61%; Warm = 18.3%; Hot = 9.33% (for ‘4G’ model, see Methods)

**Table 2:** Summary of model fit measures.

Model	Number of factors	Number parameters	∆BIC	Average max probability	Proportion correct
Full	7	756	0	0.514	0.520
-PPT (climate)	6	588	51268	0.482	0.487
-JJA (climate)	6	588	37263	0.485	0.490
-Soil Depth (fixed)	6	588	35733	0.493	0.496
-Wind (fixed)	6	588	31126	0.482	0.483
-CWD (climate)	6	588	19192	0.502	0.506
-DJF (climate)	6	588	13583	0.506	0.511
-March radiation (fixed)	6	588	10695	0.502	0.511
Climate only	4	315	94058	0.443	0.426
Fixed only	3	210	212839	0.384	0.402

Top row shows the full model with seven predictors, four climatic factors (JJA, DJF, CWD, PPT) and three fixed factors that did not vary under future climate scenarios (Soil depth, Wind speed, March radiation), with 36 parameters (linear, quadratic, 2-way interaction) for each of 21 (n-1) vegetation types. BIC value for the full model is 577096. Next seven rows show models with one factor at a time removed, sorted by decreasing ∆BIC. Last two rows show models with only the climate factors, or only the fixed factors. Average max probability is the mean value of the highest probability vegetation type in each pixel. Proportion correct is the proportion of pixels in which the maximum probability type was equal to the observed type.

The predicted frequency of vegetation types based on V_max_ exceeded observed frequencies for common types, and underestimated them for rare types (S3B Fig). The percent of pixels correctly predicted for individual vegetation types varied from 0% (interior live oak) to 80% (grassland) and generally increased with the observed frequency of each type (S3C Fig). As a baseline for comparison with the 52% correct prediction, a random assignment of vegetation types, in proportion to their observed probabilities, would be correct in 17.4% of cases ($\sum f_{i}^{2}$). Assigning all pixels to the most common type (grassland) would be correct for the 35.9% of pixels that were grassland, but wrong for all others. Stochastic assignment of vegetation types in proportion to their predicted probability in each pixel would be correct in 38.7% of pixels (mean of 100 runs). Thus, the model was substantially better than these baselines. The less-than-perfect performance of our model is not due to allocation disagreement [33], as the model correctly predicts the total area for each vegetation type based on the sum of probabilities across all pixels (a constraint in the model fitting procedure, S3A Fig).

The degree of confidence in assignment of the most likely vegetation type ranged from 0.12 to 1.0 (mean = 0.52) across locations, under the baseline conditions used to parameterize the model (S4 Fig). The distribution of values was bimodal, with a peak around 0.3 and another peak at 1.0. The latter primarily reflected mapping of grasslands with very high confidence across an east-west band of high wind velocity (compare Fig 1A, S1C and S4B Figs). The lower mode illustrates that most pixels could be assigned to more than one vegetation type with relatively high probability, reflecting the observation in the field that different vegetation types are often found in close proximity and under similar conditions. Of 231 pairwise comparisons among the 22 vegetation types, 9 pairs had correlations of modeled probabilities greater than 0.5, reflecting pairs of vegetation types that occur under similar conditions (S4 Table). Maximum and minimum pairwise correlations were 0.69 (blue oak woodland vs. blue oak/foothill pine woodland) and -0.50 (Douglas fir forest vs. grassland).

### Projected change in vegetation frequencies

**Fig 3:**
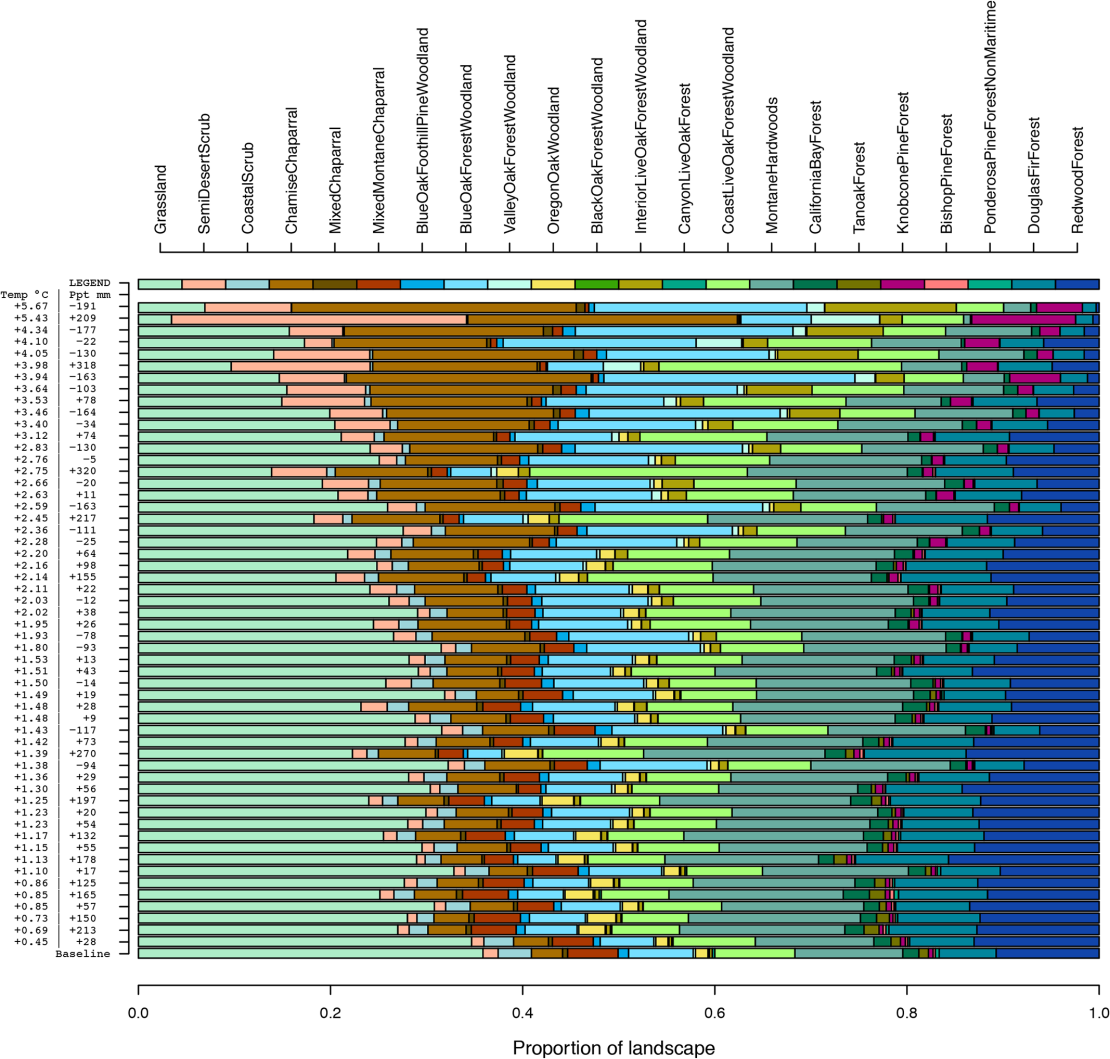
Modeled frequency of vegetation types. Relative frequency of 22 vegetation types across the San Francisco Bay Area, parameterized for the historical baseline period and then projected for alternative climates based on the recent climate (1981–2010) and 54 possible futures. Future climates are arranged in order of increased warming in mean annual temperature from bottom to top. Changes in MAT and PPT, averaged across the region for each climate scenario, are shown on the left.

**Fig 4:**
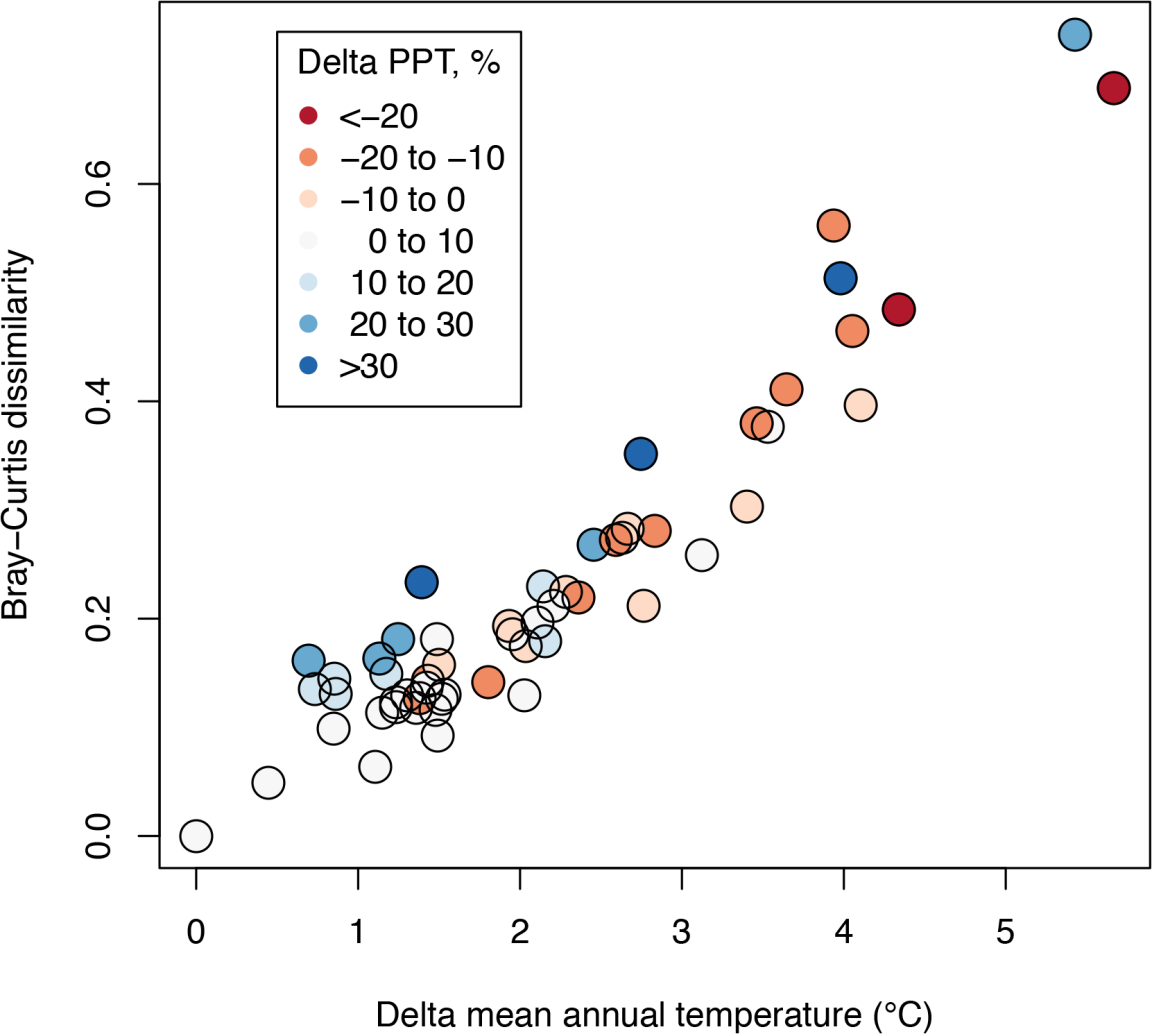
Magnitude of projected vegetation change. Bray-Curtis distance between future and baseline vegetation distributions, based on frequency distributions illustrated in Fig 3, in relation to mean annual temperature of future climates. Colors indicate change in precipitation under future climates (red = negative, blue = positive).

Projections of the PVM model across a range of future climate scenarios suggest that substantial changes in the relative frequency of the various vegetation types would be expected in response to 21^st^ century climate change (Fig 3, showing the average of relative probabilities across all pixels). Overall, under warmer and drier (e.g., higher CWD) climates, shrublands and oak woodlands expand or persist in extent, while grassland and montane and coniferous forest decline. (Results under alternative models are shown in S4 and S5 Figs see Methods). We emphasize that this is an equilibrium response model, so the model cannot address the question of how long it would take for these predicted responses to occur. The overall magnitude of change, measured as the Bray-Curtis dissimilarity index between the baseline frequency vector vs. future projections, increases linearly with increases in MAT and the absolute value of change in PPT, though temperature has a much stronger effect (multiple regression R^2^ = 0.95, Fig 4). This measure only assesses the overall frequencies of vegetation types, and not their predicted spatial locations, so it does not address the magnitude of local impacts or the projected distribution shifts of individual vegetation types (see below). Examples of projected vegetation distributions from this model, under selected future scenarios, are illustrated in Chornesky et al. [34].

### Responses of individual vegetation types

**Fig 5:**
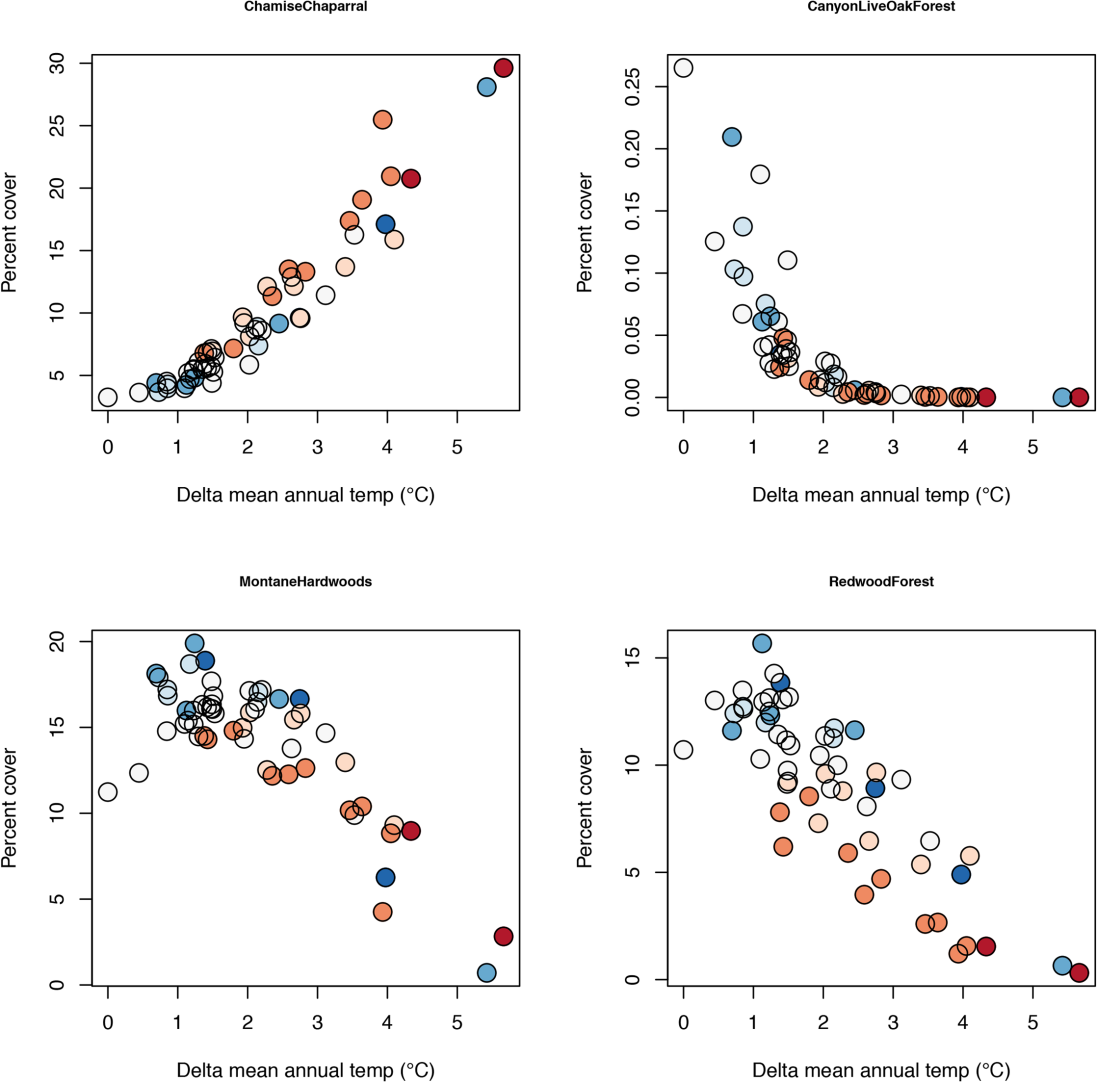
Projected change in selected vegetation types. Changes in relative abundance of four vegetation types, plotted relative to mean annual temperature (MAT) of each of the climate scenarios. Colors indicate change in precipitation (see legend in Fig 4). See S6 Fig and S5 Table for results for all 22 vegetation types.

Multiple regression was used to assess changes in landscape cover of each vegetation type, based on the sum of its predicted probability across all pixels, in response to average temperature (MAT) and PPT of future climate scenarios; CWD, DJF, and JJA all covary strongly with MAT, making it impractical to tease apart individual contributions. Non-linear relationships with MAT were evaluated, and if present either a quadratic or an exponential model was selected based on goodness-of-fit. All but one of the 22 vegetation types changed significantly with MAT: twelve declined, six increased, and three had hump-shaped responses with an initial increase followed by decline at higher temperatures (Fig 5, S6 Fig S5 Table). Vegetation types projected to increase with temperature were generally those that occupy hot, interior portions of the study region (e.g., semi-desert scrub, chamise chaparral, blue oak woodland, interior live oak woodland, knobcone pine forest). Of the vegetation types projected to decline with temperature, four exhibited negative exponential patterns reaching values close to zero in response to about 2.5°C of warming (black oak forest/woodland, canyon live oak forest, tanoak forest, and non-maritime ponderosa pine forest). Seventeen of 22 types exhibited significant responses to precipitation, nine decreasing and eight increasing at higher rainfall (and vice versa in response to lower rainfall) (S6 Fig). It is important to remember that the multinominal logistic is a zero-sum model, so if some types increase others must decrease, placing a constraint on the range of responses across the individual vegetation types.

### Changes in weighted mean indices of location

**Fig 6:**
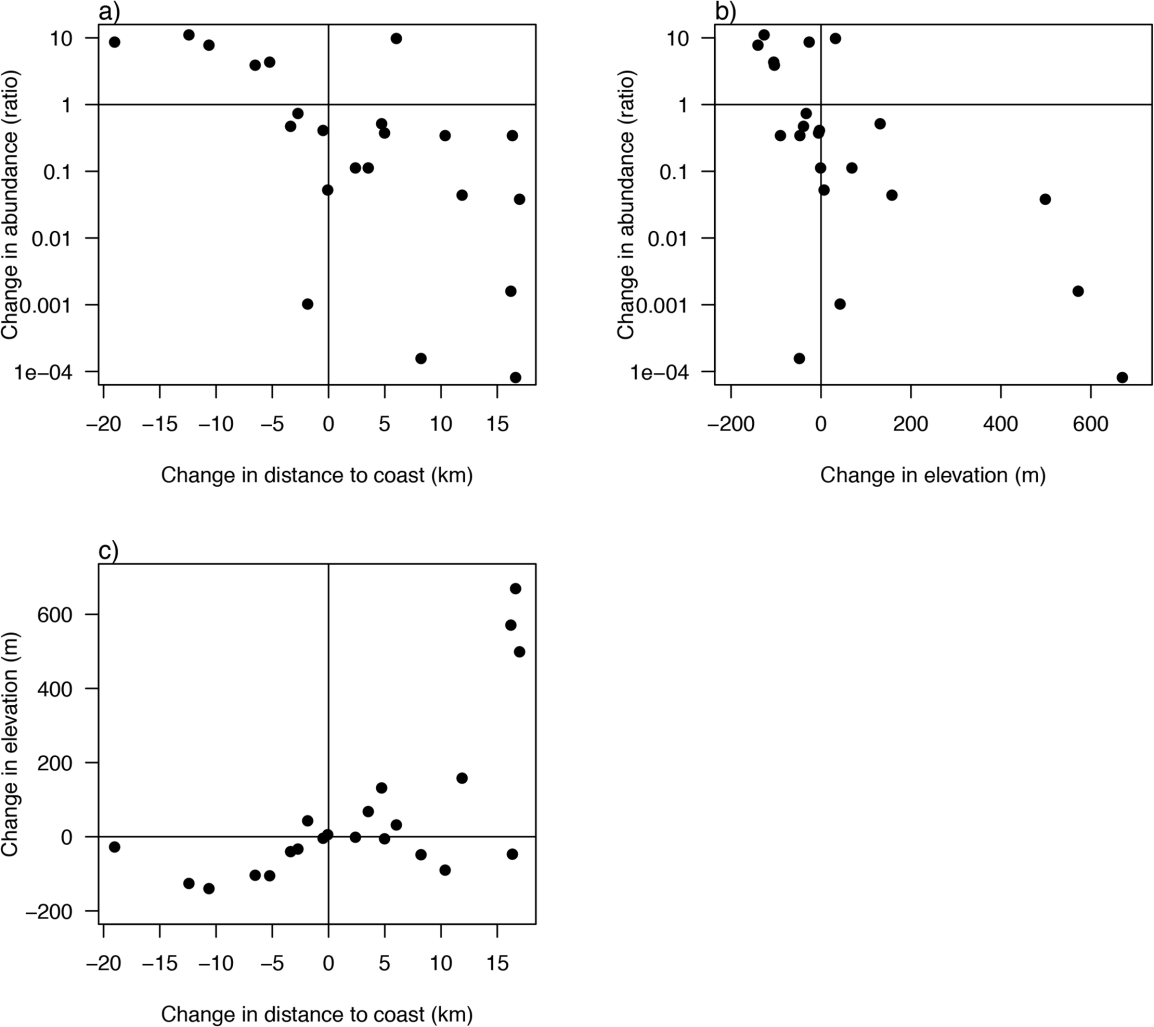
Projected changes in vegetation location and abundance. Changes in the location and abundance of the 22 vegetation types illustrated for the GFDL-A2-2070-2099 future. a) Change in distance to coast vs. proportional change in abundance (sum of relative frequencies across all pixels); b) change in elevation vs. proportional change in abundance; c) Change in distance to coast vs. change in elevation.

Changes in the spatial distribution of each vegetation type were assessed by calculating the mean elevation and distance from the coast, weighting each pixel by the probability that it was occupied by the respective vegetation type. In general, vegetation types that increased in frequency under warming climates shifted towards the coast and to slightly lower elevations and deeper soils, while declining types shifted away from the coast and uphill, and sometimes onto more exposed sites (i.e. south-facing). This is illustrated under one future scenario, GFDL-A2-2070-2099, +3.94°C MAT (Fig 6). Note that projected shifts towards the coast, or inland and uphill, can offset rising summer temperatures by moving towards cooler summer locations along these geographic or elevational gradients. In contrast, winter temperatures are warmer near the coast, so vegetation shifting towards the coast would compound the exposure to winter warming due to the combined effects of the geographic shift and climate warming.

### Spatial patterns in sensitivity of vegetation change

**Fig 7:**
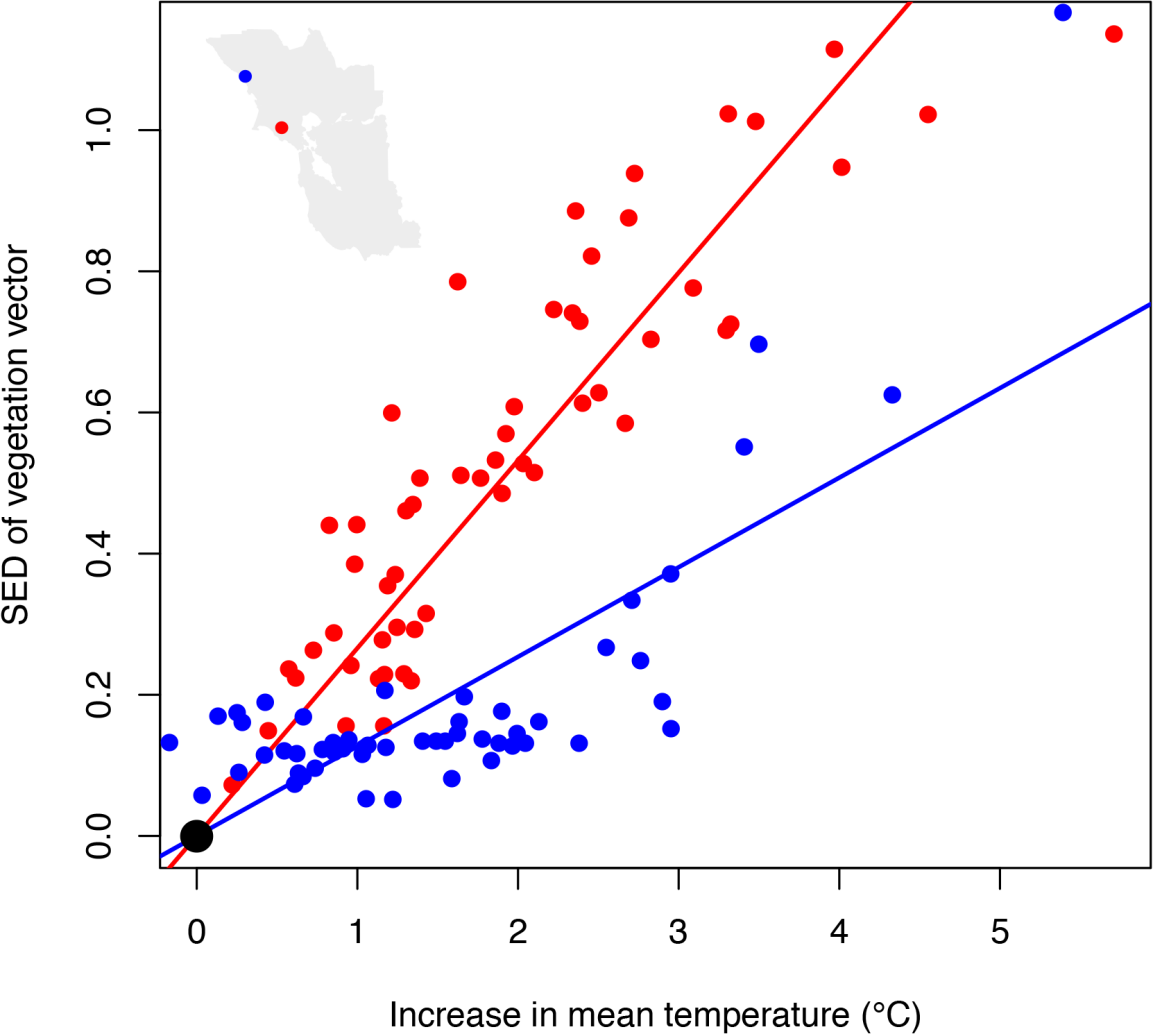
Projected vegetation change at selected sites. Illustration of Bray-Curtis distances between baseline and future vegetation vectors (similar to Fig 3) for two selected pixels across our model domain. The slope of this relationship was used as a measure of the sensitivity of projected vegetation change in relation to climate, with mean annual temperature as a proxy for changes in JJA, DJF, and CWD. Much of the scatter around each regression line represents additional effects of PPT. Red illustrates a site with high sensitivity (slope = 0.298) and blue a site with low sensitivity (slope = 0.104).

**Fig 8:**
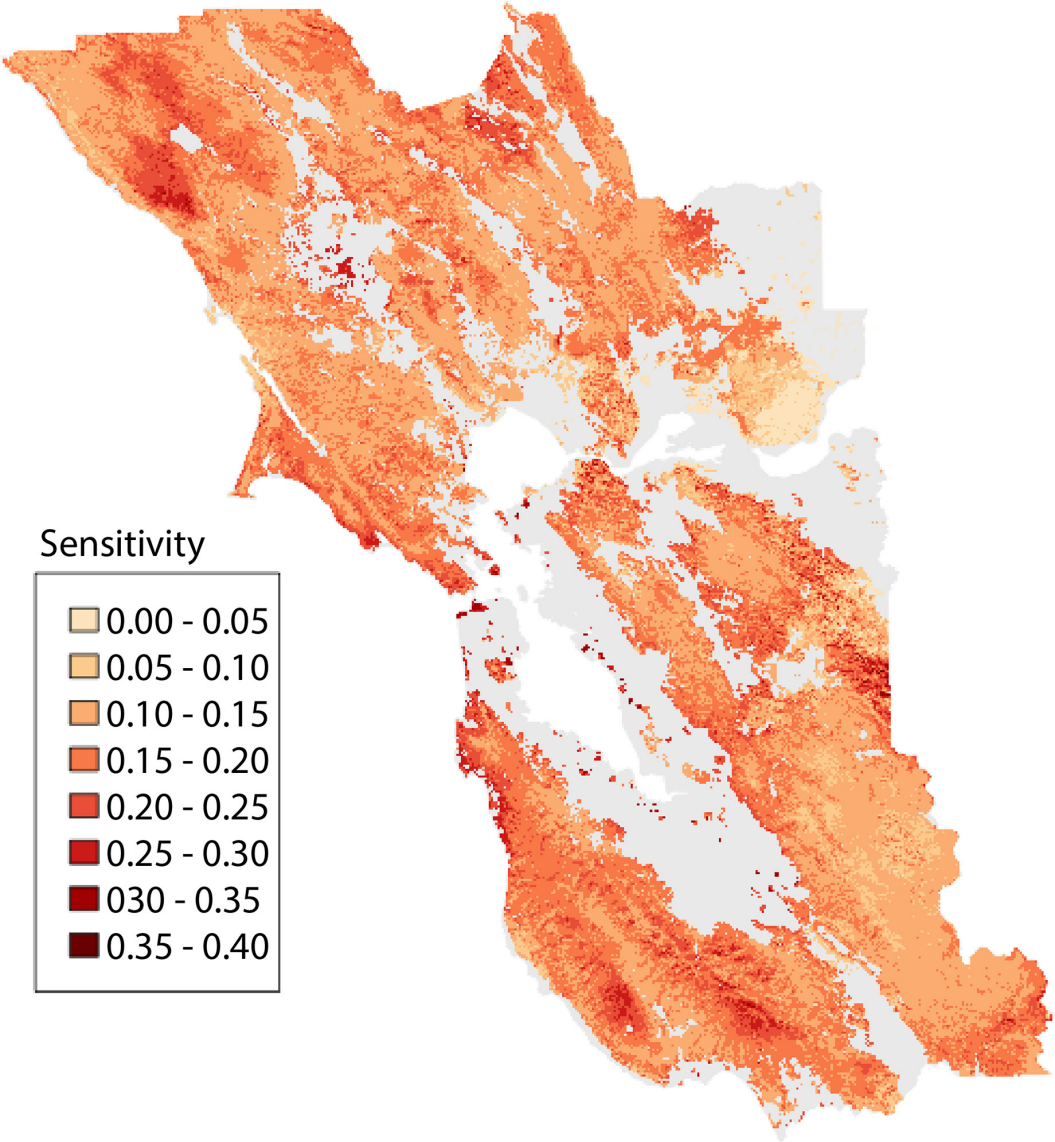
Projected sensitivity of vegetation to climate change. Map of sensitivity values, based on regression slopes for Bray-Curtis distances between baseline and future climate vs. mean annual temperature (illustrated in Fig 7).

**Fig 9:**
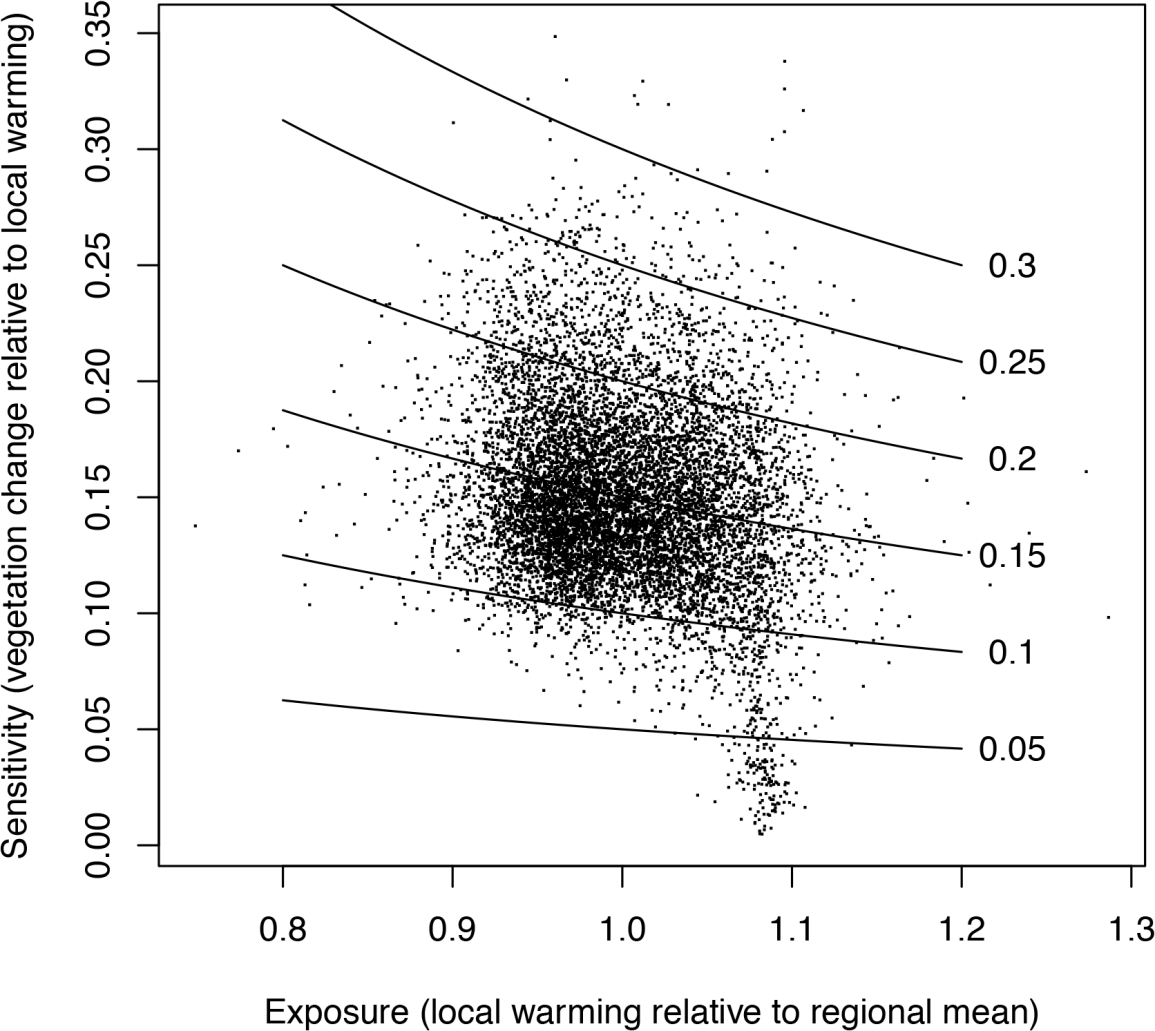
Exposure vs. sensitivity of vegetation to climate change. Variation in exposure vs. sensitivity, showing the much greater range of variation in the latter. The product of the two axes is the measure of potential vulnerability of vegetation in response to climate change, shown by the isoclines.

Analysis of the Bray-Curtis distances between baseline and future vegetation vectors for each pixel reflects the magnitude of projected change at each location across the range of future climates. Local sensitivity of vegetation to climate change was assessed using linear regression as the slope (forced through the origin) of these dissimilarity measures relative to increase in MAT in that pixel (a proxy for DJF, JJA, and CWD change). These slopes exhibited a wide range from 0.002 to 0.4 (Fig 7) and variation across the region was distributed in a fairly patchy pattern (Fig 8). The relative exposure or magnitude of climate change (slope of local change in MAT relative to the regional average) varied only slightly, from 0.8 to 1.2, with higher values along the eastern boundary of the SFBA and some coastal locations, and lower values at higher elevations and along the north coast (S7 Fig). The overall projected impact of climate on vegetation (the product of local sensitivity and exposure) was thus primarily driven by variation in local sensitivity (Fig 9), so the spatial patterns in impact were similar to sensitivity (S8 Fig).

**Fig 10:**
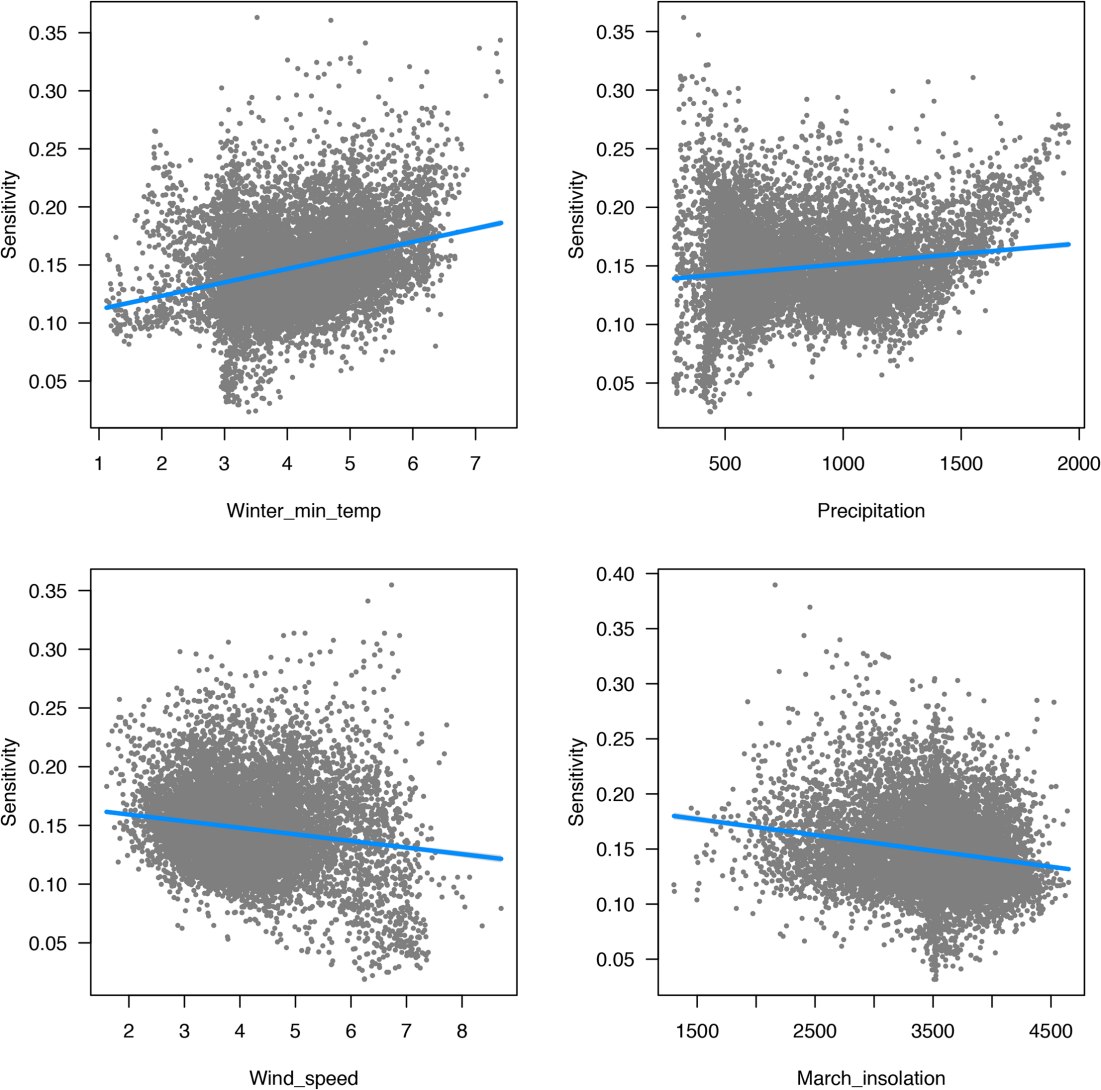
Correlates of projected sensitivity of vegetation to climate change. Scatterplots of sensitivity of vegetation to climate change (based on slopes of Bray-Curtis distances, as shown in Fig 7) vs. four landscape factors: a) DJF minimum temperature; b) precipitation; c) wind speed; d) equinox solar radiation. Light blue lines show partial regression slopes from multiple regression on these four factors.

We evaluated spatial variation in sensitivity as a function of the baseline values for the seven predictor variables using multiple regression. Across the entire region, a multiple linear regression explained 19.4% of the variation. While all variables were statistically significant (given the very large sample size), we were able to drop the three variables with the lowest contributions (soil depth, CWD and JJA), and R^2^ was reduced only slightly to 19.0%. Of the remaining four variables, the direction of the relationship with sensitivity was in some cases surprising. Vegetation was predicted to be more sensitive to climate change in sites with warmer winter (i.e. near the coast), higher rainfall, lower wind, and lower solar insolation (i.e. north-facing slopes) (Fig 10). To illustrate the effect of aspect and insolation, we selected four pairs of sites in different parts of the SFBA, with one on a north-facing site with high vegetation sensitivity, and the other on a south-facing slope with lower sensitivity. For each one, we then created an artificial gradient with MAT increasing up to 4°C, CWD, JJA, and DJF increasing proportionally, as shown in Fig 2, and PPT held constant. These graphs illustrate the greater vegetation change on the north-facing slopes, which in each case go from a high probability of one type (redwood or grassland in these cases) and rapidly shift to a high probability of another (shrubland or blue oak woodland, respectively) (S9 Fig). In contrast, the south-facing slopes have probabilities more evenly distributed over several different types, and the distribution does not change much up to 4° of warming.

## Discussion

A wide range of modeling approaches has been developed to project biotic responses to future climate change and to evaluate responses of individual species along with the overall responses of communities and ecosystems. Species distribution modeling provides a powerful approach for individual species, but it is difficult to project changes in future communities from the geographic ranges of the constituent species [8]. Modeling of dominant species that define major habitats or vegetation types, as in this paper, facilitates landscape scale analysis and provides an important connection to habitat-based conservation planning. However, such approaches either must assume that communities move together as a whole, which we know is not the case, or focus only on the dominant taxa and leave unanswered the question of how the rest of the biota will respond. We take the latter approach, and emphasize that our model is useful to evaluate possible shifts in dominant taxa that define major habitats, but consequences for associated plant and animal species are unknown.

Multinomial logistic regression has been used previously to model vegetation cover, soil maps, and land use change [35–38], but we are not aware of previous applications to model vegetation shifts in response to future climate change (Calef et al. [39] used a hierarchical logistic regression which shares some of the features of our model). The probabilistic nature of the model has advantages and disadvantages. Importantly, the probability vectors for vegetation types at each pixel acknowledge the fact that vegetation is not fully determined by climate and soils; a given set of conditions may be suitable for several different vegetation types, and the vegetation of a particular site will reflect historical factors (e.g., land use, fire, disturbance history, priority effects in community assembly) as well as deterministic factors that are not captured in the model (e.g., bedrock type). The relative probabilities also allow us to quantify the magnitude of potential change, using vector distance methods, without having to make a discrete prediction of what the present or future vegetation is at each point. This is advantageous as it protects against a false sense of precision in both the model and future projections. Moreover, as discussed above (S3B Fig), using the model to map the most likely vegetation type leads to an under-representation of rare types, which may be the ones of greatest interest for conservation planners.

A second critical feature of our approach is that it is a zero-sum model: unlike in species distribution models, the model generates a prediction for every pixel from the available vegetation types, and reductions in some types are necessarily balanced by increased probability of others. It is thus impossible to generate the prediction that a novel vegetation type from outside the region, or a novel combination of species from within the region, will emerge under future climates. It is also impossible to project expansion of invasive species, if they are not part of the baseline model parameterization (the only vegetation type dominated by exotic species in our model is grassland, composed of Eurasian annual grasses). And finally, the model does not address the extreme events or ecological mechanisms that might trigger vegetation type conversion (e.g., fire, drought, disease). We return to this point below.

Lastly, like species distribution modeling, all models that use current spatial distributions to project temporal change invoke an equilibrium assumption and a space-for-time equivalence. In other words, the vegetation of sites that will be warmer in the future is predicted based on what is observed at warm sites in the historical baseline data. As it is an equilibrium model, there are no time delays in vegetation change or constraints on change such as limited seed dispersal, or occupancy by long-lived trees (such as redwoods) [40]. Non-analog climates may also appear in the future, and the model will then extrapolate beyond the conditions under which it was parameterized. In our area, future climates include warmer winter temperatures along the coast, and warmer and drier summer conditions inland, that extend beyond the range of historical conditions. These novel conditions become fairly widespread in scenarios with MAT increases greater than 4°C, but exclusion of these scenarios did not alter the essential results of our model (results not shown). However, we would treat results around the edges of the domain with care, especially for conservation planning purposes where novel climates may be accompanied by novel species and vegetation types not considered in the model.

With these caveats in mind, what lessons are learned from this approach to modeling the future of Bay Area vegetation in response to a changing climate? The results of this model can be viewed from two perspectives: first, land-based, the approach most relevant for land managers and for those planning reserve networks, and second, species- or vegetation type-based, focused on the fate of particular vegetation types.

### Exposure, sensitivity and vulnerability of vegetation to a changing climate

We have used a range of future climate scenarios, together with our modeling framework, to decompose the potential vulnerability of vegetation to climate change into components due to exposure and sensitivity. In social sciences, a third feature of a system, the adaptive capacity, is also examined to incorporate the potential for system-level responses to offset potential impacts. In biological systems, adaptive capacity can refer to intrinsic capacity for responses via phenotypic plasticity, adaptive evolution or other biological mechanisms [23]. In the context of distribution modeling, some of these responses will be incorporated in the model, because they are expressed in the species current spatial distributions. Other factors, such as the effects of elevated CO_2_ on drought tolerance [41], are missing because they are not captured in the spatial gradients used to build the model. Societal contributions to adaptive capacity are also not incorporated, via altered conservation strategies, managed relocation, restoration projects, and the like. Factors that enhance adaptive capacity will reduce the actual long-term impacts, while other features that are not incorporated in the model, such as dispersal limitation, could lead to greater impacts (at least in the short run), due to transient, disequilibrium responses [40].

Like most of the globe, our entire study region is projected to experience warming in winter and summer temperatures. However there is a great deal of uncertainty with respect to future precipitation levels. One important result from our water balance model is that climatic water deficit (sensu [42]) is projected to increase due to rising temperatures, even for scenarios with increasing precipitation (see Fig 2). Thus, in our study region the future is expected to be effectively drier for terrestrial ecosystems (in contrast, precipitation has very strong effects on river flows and freshwater ecosystems). Within the region, the cool ocean is expected to buffer warming trends, leading to less exposure to warming close to the ocean (S7 Fig see 17). However, spatial variation in exposure is fairly low, based on the degree of change in each pixel relative to the regional mean: 2.4-fold variation in JJA, 2.0-fold in DJF and 2.0-fold in CWD. As a common metric of exposure for each site, we use changes in MAT (though it is not one of the predictor variables in the model), and change in MAT exhibits 1.5-fold variation across our region (Fig 9).

In contrast to this limited variation in exposure, we find highly variable and spatially patchy patterns in the degree of sensitivity of vegetation to climate. Sensitivity is measured as the slope of the Bray-Curtis vector distance of vegetation probabilities, comparing future scenarios to the historical baseline, versus changes in MAT as a proxy for the magnitude of climate change across future scenarios. Sensitivity values span a 200-fold range of variation, much greater than the range in exposure (Fig 9). As a result, projected vulnerability for any given patch of land is driven by sensitivity rather than exposure. Although our predicted sensitivity is very patchy, there are trends in relation to landscape position, with greater sensitivity in sites with warmer winter temperatures (i.e. closer to the ocean), less wind, higher precipitation, and lower solar insolation (i.e. north-facing slopes) (Fig 10). These results in part challenge the conventional view of ‘climate refugia’, which are often presented as locations with more extreme (cooler or wetter) or more stable conditions within a landscape (e.g., [26]). It is important to recognize that cooler or moister topoclimates may remain relatively cooler or moister relative to other landscape positions, and they may represent the last sites where cool or moist-adapted species persist within a given region. But this does not mean that the sites are climatically or biologically stable, as species distributions may shift and cool-adapted species could invade these sites and establish new populations, even as they are disappearing across the landscape. This is consistent with paleoecological evidence that only a portion of regional biota survives in refugia, and communities within refugia are expected to be dynamic over time [43]. These possibilities emphasize the importance of distinguishing in-situ refugia, where species may persist in their current locations, from ex-situ refugia (even at a topographic scale) to which species may retreat in the face of warming [18].

The greater projected sensitivity in coastal locations and north-facing slopes presents an interesting parallel to spatial patterns of beta-diversity. Given the space-for-time equivalence of this model, the projections in response to temporal change are essentially a reflection of patterns of turnover in vegetation across spatial gradients which are used to parameterize the model. While beta diversity formally is related to species compositional turnover, we believe that transitions in dominant vegetation likely reflect similar patterns, as distributions of many other taxa are often linked to dominant habitat features. Harrison et al. [44] have summarized a range of studies that document greater ‘structured’ beta diversity (or turnover, sensu [45]) across environmental gradients in more productive settings. Here, we project the sensitivity (= turnover) of vegetation in response to a hypothetical future gradient of climate change in time, and propose that there is a formal equivalence between spatial beta diversity and temporal responses of biotic communities to climate change. While we do not have direct measures of productivity, vegetation closer to the ocean and on north-facing slopes is often taller, closed-canopy conifer or oak woodlands, compared to oak savannas and chaparral in interior and south-facing slopes. If the space-for-time equivalence that we propose is correct, this suggests the counter-intuitive result of greater sensitivity to climate change in productive ecosystems, and lower sensitivity (or greater resistance) in less productive systems (see S9 Fig).

It is important to again emphasize the equilibrium nature of our model, as the projected sensitivity does not identify either the time frame of actual change, or the ecological mechanisms that will trigger vegetation type conversion or community change. Under more extreme climate change scenarios (MAT increases of 4°C or more), our model forecasts a shrubbier future for the Bay Area. Some of the projected transitions have well known mechanisms, such as succession from grassland to shrubland [46]; others, such as redwood to shrubland, have not been observed in the recent past in the absence of human disturbance. For such transitions to occur, large-scale disturbances (e.g. fire, disease or drought) may be required, and predicting the likelihood, location, and timing of these catastrophic events is a current research challenge. These ideas suggest a renewed attention to classical ideas of disturbance, succession, and vegetation change in a non-stationary climate [40].

As with most other equilibrium models, there is also no dispersal limitation on vegetation change. We believe that this is less of an issue at the local scale due to the patchiness of vegetation in our region; previous analyses of this model found that most—although certainly not all—projected transitions would require dispersal from existing patches within ≤1 km to seed new patches of projected vegetation [47]. The model does not address the potential for spread of exotic species, incursion of new species from outside our spatial domain, or novel vegetation types. If dispersal limitation and demographic constraints (e.g., slow growth) do constrain vegetation transitions, the high fecundity, dispersal and growth rates of many exotic invasives could promote rapid spread following disturbance events in a changed climate.

### Responses of individual vegetation types and dominant plant taxa

Until recently, the literature on range shifts in relation to climate change has focused on a simple model of up-slope and poleward migration in response to warming (the ‘global fingerprint’; [48,49]). Recent meta-analyses have highlighted evidence for a much more heterogeneous range of responses, recognizing that it is also very difficult to attribute historical range shifts to climate in individual cases [50,51]. Observations and projections of down-slope movement have attracted particular interest, but the possible mechanisms driving down-slope migration have engendered debate. For example, Crimmins et al. [24] suggested that many California plant species shifted downslope in the 20^th^ century, and that these changes could be attributed at least in part to increasing rainfall, allowing species to shift towards lower rainfall sites at lower elevations. However, Stephenson and Das [52] argued based on water balance theory that 20^th^ century climate changes would not lead to downhill shifts in vegetation, due to interactions with evaporative demand and soil water holding capacity.

Our model predicts downslope distribution shifts for several vegetation types, even in response to a warmer and drier future. While it is difficult to extract the specific drivers from multivariate models, we highlight two mechanisms that contribute to these patterns. First, we used a zero-sum model, so if some vegetation shifts uphill and/or contracts, other types must increase and move to occupy lower elevation sites. To some degree this may simply reflect the relative sensitivity (statistically) of different vegetation types, as one type may expand relative to another if it is relatively better suited to the changing conditions even if in absolute physiological terms the impacts may be negative. In addition, at landscape and regional scales, temperature inversions and increased moisture availability in valley bottoms invert the relationships between temperature, moisture and elevation. Contrasts between north and south slopes also offer opportunities for small-scale distribution shifts that could offset substantial climate change [53]. Near the ocean (especially where sea surface temperatures are cold), coastal-inland gradients create a regional inversion, as low elevations toward the coast are cooler in summer than higher elevations further inland. The fog belt along the California coast further enhances this effect, generating high moisture availability and cool temperatures in locations directly impacted by the marine layer (up to an elevation of about 400 m). Fog projections in response to climate change are still highly uncertain, though there is evidence of a historical decline in fog frequency in our region [54]. Our results, together with recent studies of topoclimate heterogeneity, emphasize the importance of considering climate refugia and biotic responses to climate change at multiple spatial scales, and the potential for heterogeneous responses across topographic and regional climate gradients [26,51,55].

### Conservation implications

This work was motivated by collaborations with local non-profit organizations engaged in strategic planning of open space acquisition and management for biodiversity conservation [29,34,56]. One of the clear implications of this work is the importance of conserving heterogeneous and diverse vegetation at local scales to provide propagule sources that can facilitate topographic shifts and habitat transitions. The probabilistic nature of our projections aligns with a portfolio view of the value of biodiversity, as it is difficult to know exactly which species or habitats will expand in the future. Maintaining a diversity of existing species and communities, with healthy populations to provide sufficient propagules to seed new populations, is critical in the face of this uncertainty. The analysis of sensitivity also highlights the possibility that potential climate refugia (e.g., north-facing slopes) may still exhibit substantial vegetation change in response to future climates. It is thus critical to protect a wide spectrum of habitats, environments and climatic conditions, to ensure seed sources for the ‘winners’ under future climate, as well as refugia where species threatened by climate change may persist as long as possible [57].

## Methods

### Study region

The San Francisco Bay Area (SFBA), as defined here, consists of the 9 counties that border the San Francisco Bay (Marin, Sonoma, Napa, Solano, Contra Costa, Alameda, Santa Clara, San Mateo, and San Francisco) plus adjacent Santa Cruz county. Elevations range from sea level to 1359 m, with rugged topography defined by the south-north oriented Inner and Outer Coast Ranges and intervening river valleys (S1A Fig). Climate is mediterranean-type, with cool, wet winters, and hot, dry summers. There is a strong oceanic influence, with mild winters and cool summers near the ocean, and a sharp gradient towards hotter summers inland; average summer maximum temperatures increase from about 15°C at the coast to >35°C inland (equivalent to descending a 3000 m peak), while winter minimum temperatures exhibit the opposite gradient and decrease about 6°C from coast to inland (Fig 1B and 1C). Substrate and soil types are heterogeneous, with isolated outcrops of serpentine soils that support distinctive plant communities (not considered in this study). Soil depth also varies greatly, influencing water balance (S1B Fig see below). Wildfire is relatively infrequent, though there is a long history of fire since the arrival of Native Americans approximately 10–12,000 years ago, and the role of fire shaping modern vegetation in this region is poorly understood.

Vegetation of the SFBA consists of a complex mosaic of vegetation types, including herbaceous grasslands, shrublands, fire-prone chaparral, deciduous and evergreen oak woodlands, mixed hardwood and coniferous forests (Fig 1A). The distribution of vegetation types is influenced by edaphic, topographic, and climatic factors. In many locations, distinct vegetation types occur in close proximity, suggesting broad overlap in climatic tolerances. Which type is observed depends on substrate and topography, and historical factors, including fire, human land use (especially logging and grazing), herbivory (especially black-tailed deer), and disease (e.g., sudden oak death, *Phytophthora ramorum*). The vegetation of the Bay Area has responded dynamically to climate change in the past, including the rapid warming following the last glacial maximum and the Holocene altithermal [58,59], and has been impacted by human activity since the arrival of Native Americans more than 10,000 years ago, and even more dramatically by European-Americans since the 18^th^ century [60]. Here we focus on environmental predictors of contemporary vegetation distributions, though we recognize that these historical influences have played a significant role in shaping the modern landscape.

### Vegetation layer

We used a detailed vegetation map for the SFBA produced as part of a regional conservation planning process (the Upland Habitat Goals Project; [29]). The map was based on remote sensing, ground truthing and vegetation plot data sets, and adjustments by expert opinion [29]. The native map resolution is at a 30 m pixel scale, with 63 cover classes. We chose 25 natural and semi-natural vegetation types to include in our modeling effort, covering 61% (1.2 million ha) of the terrestrial area (Table 1 and S1 Table). Of the remaining area, 12 cover types included urban and agricultural areas, non-native vegetation, water, and rock, and an additional 26 native vegetation types were excluded that are either very rare (<2000 ha each) or occur on special edaphic conditions (sand dunes, serpentine outcrops, riparian zones). All of our selected vegetation types are dominated by native taxa, except grasslands which are almost entirely composed of Eurasian annuals that established in the 18^th^ and 19^th^ centuries. Different grass species are prevalent across the coastal-inland climatic gradient, but field data are not available to comprehensively map the floristics of these communities. Instead, grasslands in the original vegetation map were subdivided by climatic zones based on summer maximum temperature thresholds (cool, moderate, warm, hot). Because of the potential circularity in modeling vegetation types that were defined by climate zones, we treated grasslands in two different ways for our model. In the ‘1G’ model, the four grassland types were collapsed into a single ‘grassland’ vegetation type for analysis; this analysis is the basis of all results presented in the main paper. Alternatively, in the ‘4G’ model, we used the four original grassland types stratified by temperature, recognizing that there is circularity in the model fitting process because they are defined based on climate. In addition, we conducted a ‘0G’ analysis in which grasslands were omitted from the model, and the distribution of woody vegetation was projected across the entire domain. The rationale for this last analysis is that much of the grassland vegetation is anthropogenic, maintained by grazing and management burning (though some grassland would probably occur without human influence, especially on coastal bluffs and dry interior hills, due to edaphic factors, natural grazing and fire). The ‘0G’ model is an estimate of the proportional representation of woody vegetation types only, if all natural lands were allowed to undergo succession. Results for the ‘0G’ and ‘4G’ models are shown in (S5 Fig).

The 1G model used a total of 22 vegetation types, with one for grassland, five shrubland, five deciduous oak or mixed deciduous/conifer woodland, six evergreen hardwood or mixed evergreen/conifer, and five coniferous forest types (Fig 1A, Table 1). One important aspect of the vegetation types in the Bay Area is that 16 of the 22 types are defined by one or two dominant woody species (e.g., ‘blue oak woodland’). The map contains no information about subdominant species, so vegetation types in this analysis can be thought of as the areas where a particular shrub or tree species or combination of a few species are dominant. The model does not make any projections about current or future distributions of subdominant taxa that may occur in these vegetation types, and we do not assume that these communities will shift as a unit in response to future climates. It also does not translate directly to a species model, as individual taxa will occupy several vegetation types as dominant and/or subdominant components (e.g., *Quercus kelloggii* is a constituent of Montane Hardwoods, as well as the namesake Black Oak Forest and Woodland).

### Predictive layers–topography, climate and water deficit

We used seven predictive layers to develop our model, four for climate and water balance (which varied under future climate scenarios, Fig 1B–1E) and three topographic and edaphic variables that were kept constant (equinox insolation, reflecting slope and aspect; soil depth; and average wind speed) (S1A–S1D Fig). We chose a limited set of climate layers to minimize the danger of overfitting the model, and reduce computational time. The topographic variables reflect features that are expected to influence plant distributions, and their incorporation in the model makes the projections of future change more conservative, as these features do not change [61]. We recognize that wind speed is likely to be impacted by climate change, so treating this as a constant variable assists in modeling current vegetation, but does not allow it to contribute as a driver of vegetation change. All summary statistics for spatial variation in climate, and for change across future scenarios, were calculated across a set of 1 million pixels randomly sampled from the areas occupied by the 22 vegetation types, as described below.

#### Soil depth

Minimum soil depth (mapped at 270 m) was obtained from the SSURGO spatial database [62], and used as a predictive factor on its own as well as an input to the water balance hydrologic model (S1B Fig). Across our spatial domain, mean soil depth = 0.9 m, with a range from 0.1–4 m.

#### Wind speed

We obtained mean annual average wind speed (m s^-1^) mapped at a resolution of 200 m [63]. Wind was incorporated due to its potential importance in determining coastal vegetation patterns as well as effects on ridge tops and other exposed locations [64]; as noted above we were not able to incorporate projections of altered wind speed under future climate scenarios (S1C Fig). Across our spatial domain, mean wind speed = 4.18 m s^-1^, with a range from 1.25–9.06 m s^-1^.

#### Solar radiation

We used a 1 arc-second (~30 m) DEM to generate a map of monthly solar radiation, accounting for solar tracks and hill shading (solar radiation module in *Spatial Analyst*, *ArcGIS*, ESRI, Redlands, CA). The layer for March was used as a predictive factor to represent the spring and fall equinoxes, capturing the strong effects of slope and aspect during both the spring growing season and the late summer dry season (S1D Fig). Across our spatial domain, mean insolation = 3475 Wh m^-2^, with a range from 437–4783 Wh m^-2^.

#### Climate

We used regional climate data originally derived from the PRISM climate project (800 m pixels) [65], and further downscaled to 270 m resolution by spatial gradient and inverse distance squared interpolated regression (the Basin Characterization Model, BCM; [31]). After model selection, three climate layers were retained in the analysis: winter minimum temperature (DJF = average minimum for December, January and February, Fig 1B), summer maximum temperature (JJA = average maximum for June, July and August, Fig 1C), and mean annual precipitation (PPT, Fig 1D) [65]. Historical climate values from the 1951–1980 period were used for model parameterization, as these reflect an appropriate baseline before the modern era of climate change [66] and vegetation distributions largely reflect the action of past climates (especially for long-lived plants). Data for the recent historical period (1981–2010) were also obtained to represent the recent period of modest warming.

#### Climatic water deficit

A landscape water balance module of the BCM, incorporating temperature, slope and aspect effects on potential evapotranspiration, monthly rainfall, and estimates of soil water storage, recharge and runoff, was used to estimate annual climatic water deficit (CWD, sensu [42]), the seasonally integrated excess of potential evapotranspiration relative to water availability [30]. CWD values were calculated at 270 m, and the same 1951–1980 baseline period was used for model parameterization (Fig 1E).

Summary statistics for climate and topographic variables are presented in S2 Table, based on the spatial domain of the model (excluding urban, agricultural and other areas). Pairwise correlations among predictors exhibited a maximum magnitude of R = -0.7 (R^2^ < 0.5) between PPT and CWD, and correlations of 0.62 and -0.61 for JJA with CWD and DJF, respectively (S2 Fig). While these are fairly strong, they are lower than the threshold of |R|>0.8 for excessive collinearity in statistical modeling [67]; further reduction in the number of predictor variables would weaken the interpretation of the future projections, especially because these spatial correlations may be different from the temporal correlations of change across time (see discussion of model selection below).

### Future climate layers

Thrasher et al. [68] downscaled the outputs of 92 climate models from the CMIP3 and CMIP5 data sets to 800 m over California using the Bias-Correction Spatial Disaggregation algorithm, with PRISM historical climate as the spatial baseline. Mean annual T_min_, T_max_ and PPT were then obtained for the SFBA and evaluated through a multivariate clustering algorithm to identify sets of models with similar magnitude and pattern of change; one model from each of 14 clusters was chosen to capture the range of possible futures for the region [69]. In addition, the GFDL and PCM models under the B1 and A2 scenarios were added to this set, as they have been used extensively in previous studies of climate change in California [16].

Downscaled monthly outputs for 2010–2100 for each of these 18 models were processed using the BCM to further downscale to 270 m and generate climatic water deficit layers ([30]; see above). Thirty year means were then calculated for JJA, DJF, PPT and CWD for 2010–2039, 2040–2069, and 2070–2099. Together with the historical data above, this resulted in 56 sets of possible climates for the SFBA which were used for projections of vegetation distributions (18 model/forcing scenarios * 3 time periods = 54 futures; + 2 historical periods; S3 Table). Mean annual temperature (MAT) was calculated as the mean of monthly T_min_ and T_max_ and used as a standardized measure of change to array scenarios along an axis of increasing warming (MAT was not used in the predictive models).

Across the 54 future scenarios, increases in MAT range from 0.69°C (for GISS-RCP2.6-2070-2099) to 5.67°C (MIROC-ESM-RCP8.5-2070-2099) S3 Table). JJA and DJF generally increased in parallel, with slightly higher increases projected in summer temperatures (Fig 2A and 2B), unlike the record of greater winter warming in the Northern Hemisphere during the 20^th^ century ([70]; see [50] for California). Changes in precipitation ranged from a decrease of -191 mm (-23%) to an increase of 320 mm (+38%), reflecting the continuing uncertainty about future precipitation trends at mid-latitudes [1] (Fig 2D). CWD increased across almost all scenarios (Fig 2C), due to the stronger effect of warming temperatures on potential evapotranspiration and summer water deficits, whereas changes in PPT primarily impact winter runoff [30].

As discussed below, we view each of these climate futures as a distinct scenario regardless of the time period, model or emissions scenario that was used to generate the original climate projections. The reason for this is that vegetation takes hundreds to thousands of years to respond to significant periods of climate change, due to limited dispersal rates and demographic time lags [40]. As with other statistical distribution modeling that uses space-for-time substitutions, the projections from this model represent the predicted equilibrium response of vegetation to a future climate [6], and do not address the transient dynamics or the time required to reach the new equilibrium [40]. Thus, the projected responses to a climate scenario generated for 2040–2069, for example, do not represent the vegetation response expected by that time; consequently, we examine vegetation responses solely as a function of the magnitude of climate change, and do not place any time frame on the projections.

### Probabilistic vegetation model

Logistic models are widely used in ecology [71], including for vegetation modeling [39]. In the conventional case there are two possible outcomes [72], which is limiting in an ecological setting in which there are more than two possible vegetation types. Here we use an extension of this statistical theory called multinomial logistic regression to build a probabilistic vegetation model (PVM). These models only differ from traditional logistic regression in that they allow *n* possible outcomes for every pixel; although these models are computationally intensive, they have many applications and have been thoroughly studied [72,73]. The structure and goal of the predictive part of the model is identical to traditional logistic models. PVM estimates multiple probabilities, with the constraint that there is only one possible outcome, so the vector of probabilities must sum to one. The maximum likelihood fit of the model maximizes the percent of values that are correctly predicted (i.e. the type with the maximum probability corresponds to the observed value), under the constraint that the sum of relative probabilities across all points must equal the actual relative frequencies of each type.

Analogous to the general form of species distribution modeling [74], this statistical framework can use any shape function to model the relationship between climate and the likelihood of a given vegetation type. We used the seven predictive layers discussed above as inputs, with linear, quadratic and interaction terms for each predictor. Spatial autocorrelation in the predictive layers and the vegetation map was not incorporated in the model fitting process. We randomly sampled 10^6^ points from the full coverage of the 22 vegetation types described above (the least common type, Black Oak Forest/Woodland was represented by 1449 points in this sample). We then sampled the seven predictor layers in their original resolution and projection using bilinear interpolation to the midpoint of the vegetation pixels. We used a maximum conditional likelihood fitting of the coefficients [75]. The model was fit with a subsample of N = 2x10^5^ pixels, to reduce computational time, and then projected for analysis of historical and future distributions to the full set of 10^6^ points. The observed fit was highly repeatable with different random samples of the vegetation layer down to N = 10^5^. At these sample sizes, performance statistics for the model [33] are identical for randomly selected training and test datasets, suggesting the PVM model is not over-parameterized [76]. Because of the large sample sizes and the risk of over-parameterization, we have used BIC for model evaluation, which at large sample sizes has a larger penalty for additional terms compared to AIC [76] (in practice ∆AIC and ∆BIC gave identical interpretations). We evaluated ∆BIC for each predictor layer, to determine relative contributions of each predictor. All analyses were conducted in *R* [77], using libraries *nnet* [75], *sp*, *maptools*, *raster* [78], and *visreg*.

### Model outputs

The predictions of the model are in the form of a 10^6^ x 22 matrix of probabilities (*p*_*i*,*j*_), where each row represents the vector of relative probabilities for the 22 vegetation types for that location on the landscape. As noted above, the sum of the relative probabilities for each type across all pixels ($f_{i}=\sum_{i}p_{i,j}$, i.e. the column sums) is constrained to equal the observed probabilities across the entire domain, under the baseline conditions used to parameterize the model (see S3 Fig). A prediction of the vegetation type for each pixel (*V*_*max*,*i*_) can be obtained based on the vegetation type that has the maximum value of *p*_*i*,*j*_ in each row (*p*_*max*,*i*_). In situations where some types are more common than others, as observed here, the multinomial logistic tends to overestimate the frequency of common types and underestimate rare types based on the discrete predictions for *V*_*max*_ (see Results, S3 Fig). This leads to the rather paradoxical situation in which we can correctly predict the relative frequency of vegetation types (*f*_*j*_) but we cannot map the location of the rare types as they will only be identified as the highest probability type in a small number of locations, or possibly not at all. For this reason, we focus our analyses of the model projections on changes in the relative frequency of the different vegetation types (across the entire region or in smaller subregions), and analysis of spatial shifts in mean position of each type, weighted by their probability across all locations; we do not recommend high resolution mapping of the predicted vegetation types, especially for conservation purposes, due to the biased representation of rare vs. common types.

For each pixel, *p*_*max*,*i*_ offers a measure of the degree of confidence for assignment of the corresponding vegetation type. These values were assessed across the spatial domain and analyzed by vegetation type to determine which types and which locations are predicted with the greatest confidence. Comparisons of relative probabilities among vegetation types also provide a measure of which types exhibit positive, zero or negative covariance in their predicted distributions, and thus which types have similar or contrasting distributions.

### Quantifying impact, exposure, and sensitivity to change

Future vegetation probabilities were predicted for the historical (1951–1980) and recent (1981–2010) periods and each of the 54 future climate scenarios. In each case, we obtain a full *p*_*i*,*j*_ matrix, and the predicted maximum probability type for each pixel (*V*_*max*_). One approach to quantify the degree of change between the baseline and future projections is to calculate the percent of pixels in which *V*_*max*_ changes, and a transition matrix could be calculated for predicted changes among all types. However, as noted above *V*_*max*_ values are biased in favor of common types, so this approach would have provided a biased estimate of projected changes. As an alternative that does not require assignment of pixels to a specific vegetation type, a continuous measure of change for each pixel was calculated based on the dissimilarity of the probability vectors for the 22 vegetation types between baseline and future climate scenarios (*D*_*b*,*f*_). Here, we use Bray-Curtis dissimilarity which is well suited to detect underlying multivariate ecological gradients [79,80], is bounded between 0 and 1, and has a useful linear behavior for these type of data [81]. We define present-to-future dissimilarity as the climate change impact, measured by this continuous dissimilarity metric without committing to specific predictions of *V*_*max*_ under the different scenarios. The Bray-Curtis difference was also calculated for the summed vegetation frequencies across the region to quantify the overall magnitude of the predicted shift in relative frequency of different vegetation types (without considering changes in spatial distributions).

The projected frequency of each vegetation type (*f*_*j*_) across the region was calculated as the column sum of the projection matrix under historical and future climate scenarios. The projected impact of climate change on each vegetation type was then evaluated by multiple regression of *f*_*j*_ relative to changes in MAT and PPT; changes in DJF, JJA and CWD are strongly correlated across future scenarios (Fig 2), so no attempt was made to separate their effects. Based on visual inspection and model fitting, linear, quadratic or exponential responses to MAT were selected. Shifts in the projected distribution of each vegetation type on geographic and topographic gradients can be calculated based on the weighted averages of the corresponding spatial layer, with the projected probability of the vegetation type in each pixel as the weighting factor. Changes in topographic measures (mean elevation, distance to coast, solar radiation, and soil depth) reflect shifts in predicted geographic distribution of vegetation types at local and regional scales.

The projected impact of climate change on vegetation in each pixel was calculated as the product of the sensitivity to local change times the magnitude of local change relative to regional means. Sensitivity to change was estimated as the slope of the regression of *D*_*b*,*j*_ values (Bray-Curtis vector dissimilarity) vs. the magnitude of local (pixelwise) climate change, across all 56 climate scenarios, with the regression forced through the origin; only linear regression was used so the slope could be interpreted as a measure of sensitivity, though responses were somewhat non-linear in some locations. Mean temperature (MAT) was used as the measure of change, as combined temperature and CWD effects were much stronger than precipitation (see results). Locations with a steeper slope indicate that the predicted vegetation changes more with respect to a given degree of climate change. The magnitude of local climate change, relative to the regional average, was calculated from a regression of MAT in each pixel vs. MAT averaged across the entire spatial domain. Pixels with a slope < 1 are warming slower than the regional average and vice versa for pixels with a slope > 1. The overall impact of future climate scenarios on vegetation at each location was then calculated as the product of sensitivity to local change and magnitude of local change (exposure). It is important to note that these measures of sensitivity, magnitude and impact capture the projected responses across all 54 future scenarios, and therefore do not depend on the selection of any one climate model or forcing scenario, and also do not use an average response across scenarios as in an ensemble approach.

Finally, we evaluated which factors contribute to spatial variation in the sensitivity of the projected vegetation changes in response to climate. Variation in local sensitivity was analyzed with respect to geographic and topographic gradients, baseline climate values, and among vegetation types using linear models. These analyses addressed the central hypothesis that cool or moist sites (north-facing slopes, deep soils, coastal locations) may be buffered from the impacts of climate change and serve as in-situ refugia for the vegetation currently in those locations.

## Supporting Information

S1 Fig Maps of topographic factors. (PDF)

S2 Fig Pairwise relationships among predictor variables, distance to coast and elevation. (PDF)

S3 Fig Observed vs. modeled frequencies of vegetation types. (PDF)

S4 Fig Distribution and map of probabilities of most probable vegetation type. (PDF)

S5 Fig Modeled frequencies of vegetation types for alternative grassland models. (PDF)

S6 Fig Relative abundance of each vegetation type across climate scenarios. (PDF)

S7 Fig Geographic variation in relative rate of increase in MAT. (PDF)

S8 Fig Geographic variation in projected impact of climate change on vegetation. (PDF)

S9 Fig Projected sensitivity of vegetation on north-facing vs. south-facing slopes. (PDF)

S1 Table List of vegetation types with scientific names of dominant species. (PDF)

S2 Table Mean and range of environmental parameters. (PDF)

S3 Table Mean of climate parameters across all climate scenarios. (PDF)

S4 Table Pairwise correlations of modeled frequencies for selected vegetation types. (PDF)

S5 Table Multiple regression of modeled abundance of vegetation types vs. climate. (PDF)
