# Supplementary figures and images for: A Geographic Mosaic of Climate Change Impacts on Terrestrial Vegetation: Which Areas Are Most at Risk?
Source: PLoS One. 2015 Jun 26;10(6):e0130629. doi: 10.1371/journal.pone.0130629 (PMC4482696; doi:10.1371/journal.pone.0130629)

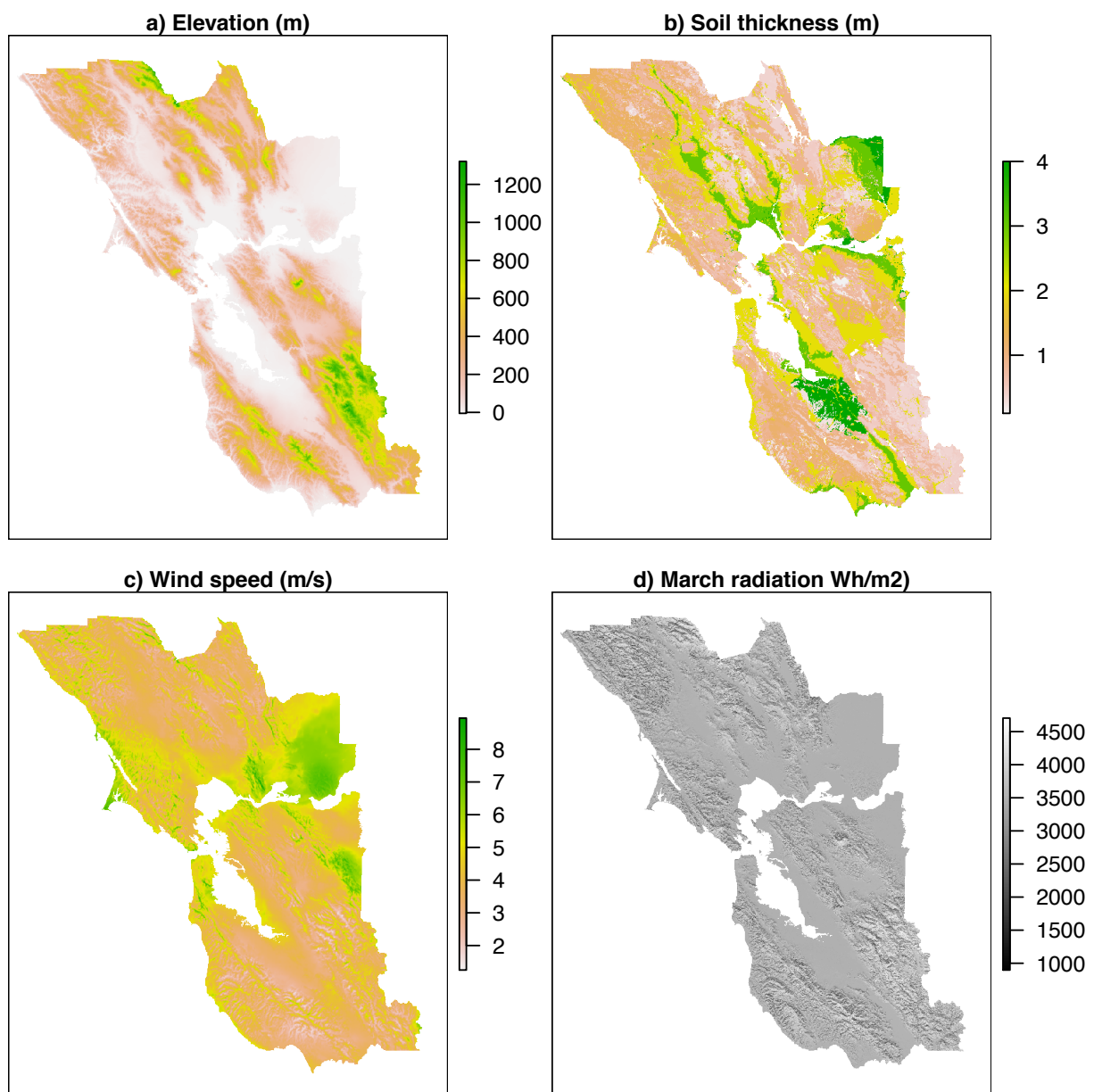

**S1 Fig. Maps of topographic factors.** (a) Elevation, (b) soil depth, (c) wind, (d) spring solar radiation.

Supplement: S1 Fig — (PDF) [file pone.0130629.s001.pdf]
