# Supplementary material for: A Geographic Mosaic of Climate Change Impacts on Terrestrial Vegetation: Which Areas Are Most at Risk?
Source: PLoS One. 2015 Jun 26;10(6):e0130629. doi: 10.1371/journal.pone.0130629 (PMC4482696; doi:10.1371/journal.pone.0130629)
Supplement: S2 Fig — (PDF) [file pone.0130629.s002.pdf]

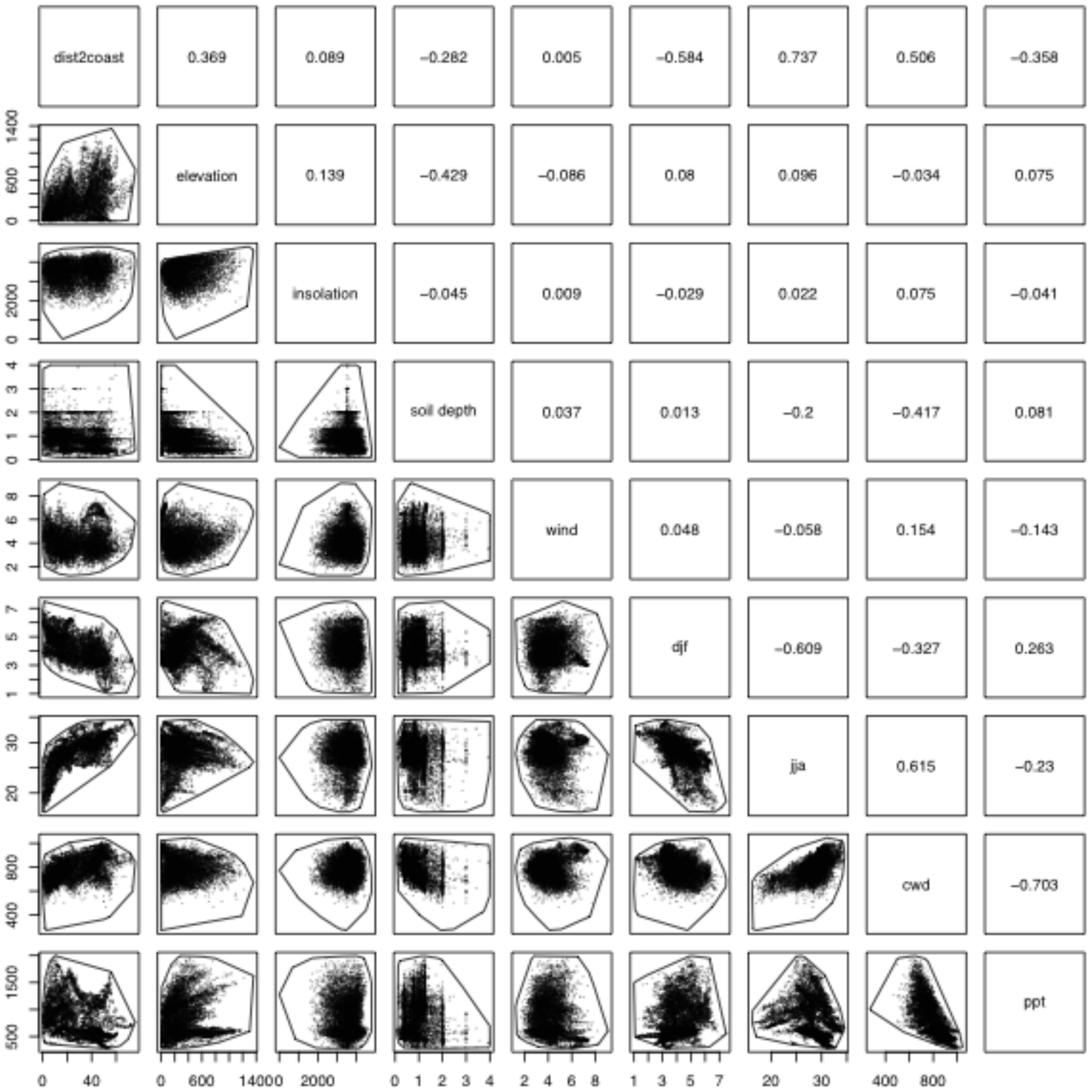

S2 Fig. Pairwise relationships among predictor variables, distance to coast (dist2coast) and elevation (dem). Plots are based on 10,000 random points and convex hull show full coverage for all 1 million points. Upper diagonal lists correlation coefficients.
