# Supplementary material for: A Geographic Mosaic of Climate Change Impacts on Terrestrial Vegetation: Which Areas Are Most at Risk?
Source: PLoS One. 2015 Jun 26;10(6):e0130629. doi: 10.1371/journal.pone.0130629 (PMC4482696; doi:10.1371/journal.pone.0130629)
Supplement: S3 Fig — (PDF) [file pone.0130629.s003.pdf]

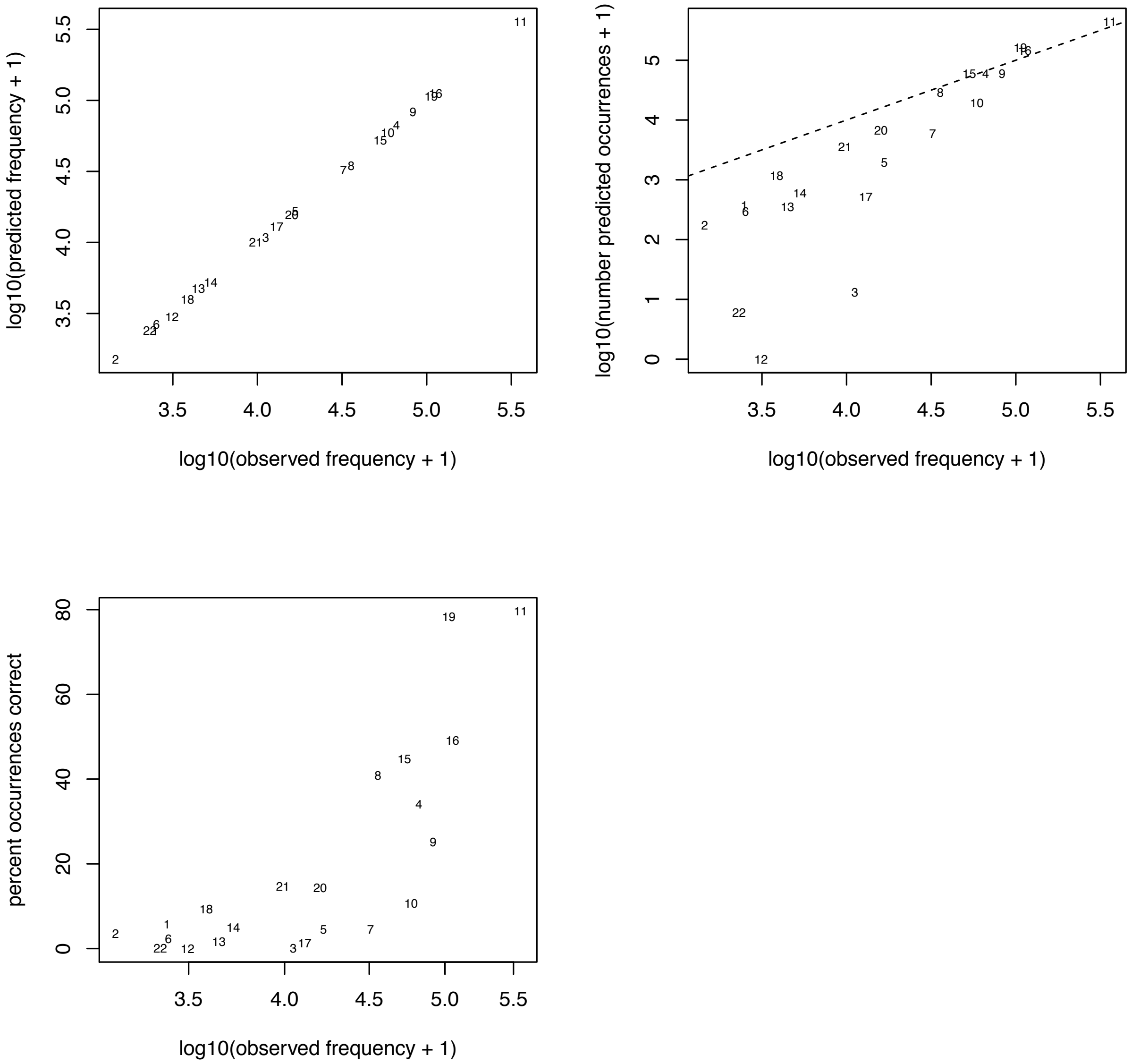

S3 Fig. Relationships between observed and modeled frequencies of each vegetation type. a) Observed vs. predicted based on sum of relative frequencies. b) Observed vs. predicted based on number of pixels in which a vegetation type had the highest probability (Vmax). c) Observed vs. the percent of observed sites correctly predicted based on Vmax.
