# Supplementary material for: A Geographic Mosaic of Climate Change Impacts on Terrestrial Vegetation: Which Areas Are Most at Risk?
Source: PLoS One. 2015 Jun 26;10(6):e0130629. doi: 10.1371/journal.pone.0130629 (PMC4482696; doi:10.1371/journal.pone.0130629)
Supplement: S4 Fig — (PDF) [file pone.0130629.s004.pdf]

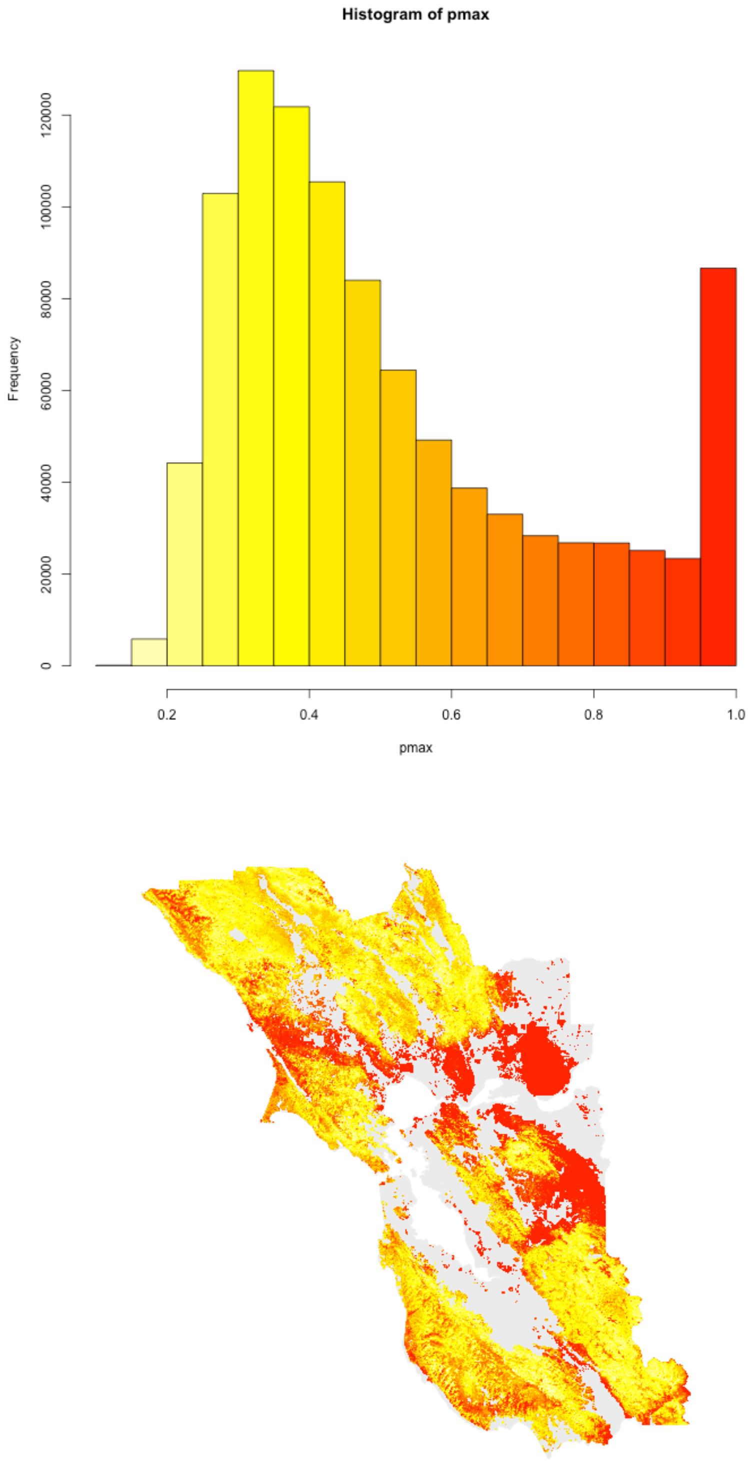

S4 Fig. Modeled probabilities of most likely vegetation type. A) Distribution of p_{max} values (probability of the most probable vegetation type). B) Map of p_{max} values.
