# Supplementary material for: A Geographic Mosaic of Climate Change Impacts on Terrestrial Vegetation: Which Areas Are Most at Risk?
Source: PLoS One. 2015 Jun 26;10(6):e0130629. doi: 10.1371/journal.pone.0130629 (PMC4482696; doi:10.1371/journal.pone.0130629)
Supplement: S5 Fig — (PDF) [file pone.0130629.s005.pdf]

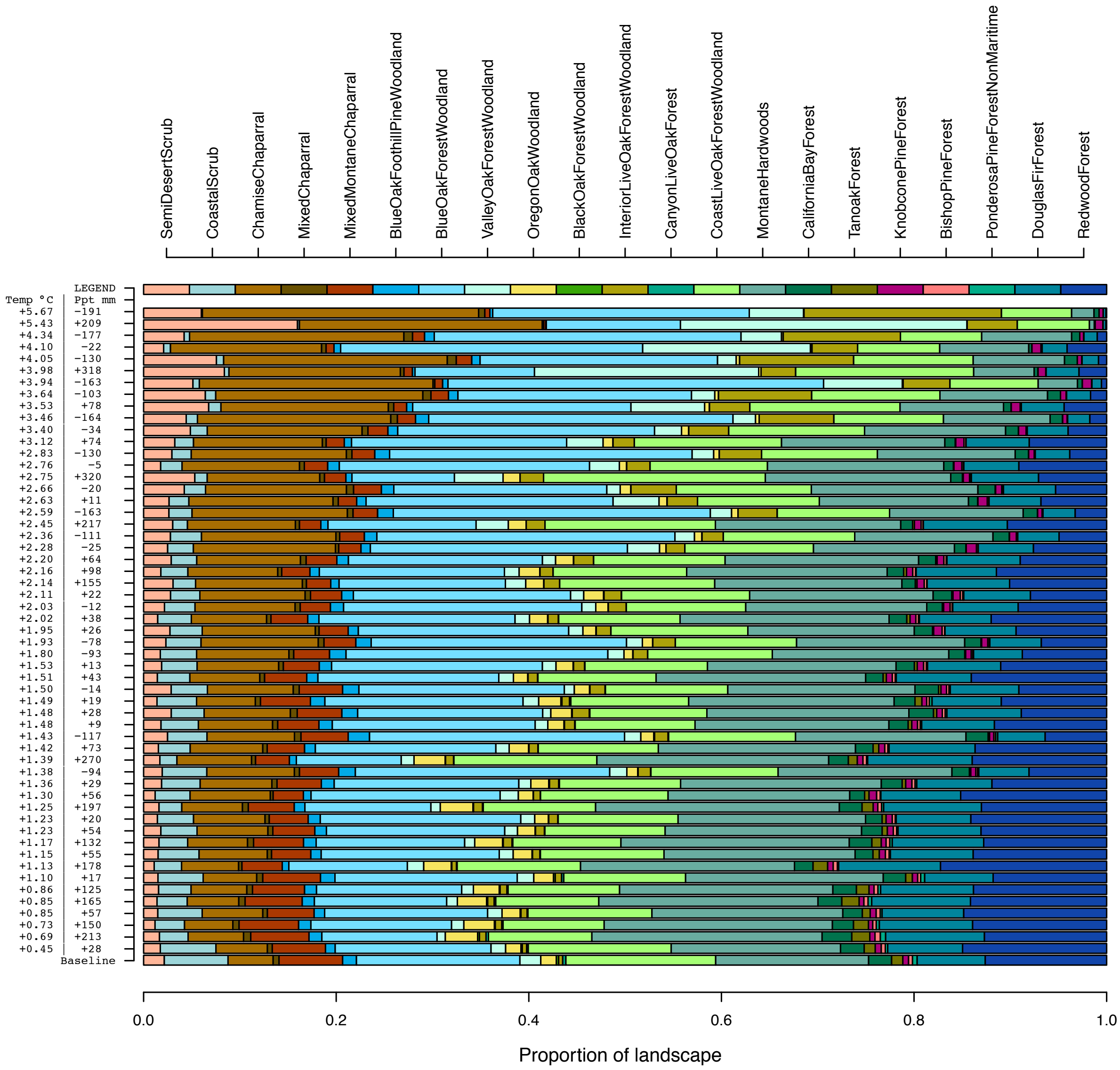

S5 Fig. Modeled frequencies of vegetation types for alternative grassland models. a) '0G' model, fit and projected for woody vegetation types only. Relative shifts are similar to the '1G' model, after accounting for removal of grassland. In general, warmer and drier climates lead to increased dominance of shrublands and blue oak forest/woodland, and reductions in montane hardwoods and coniferous forests.

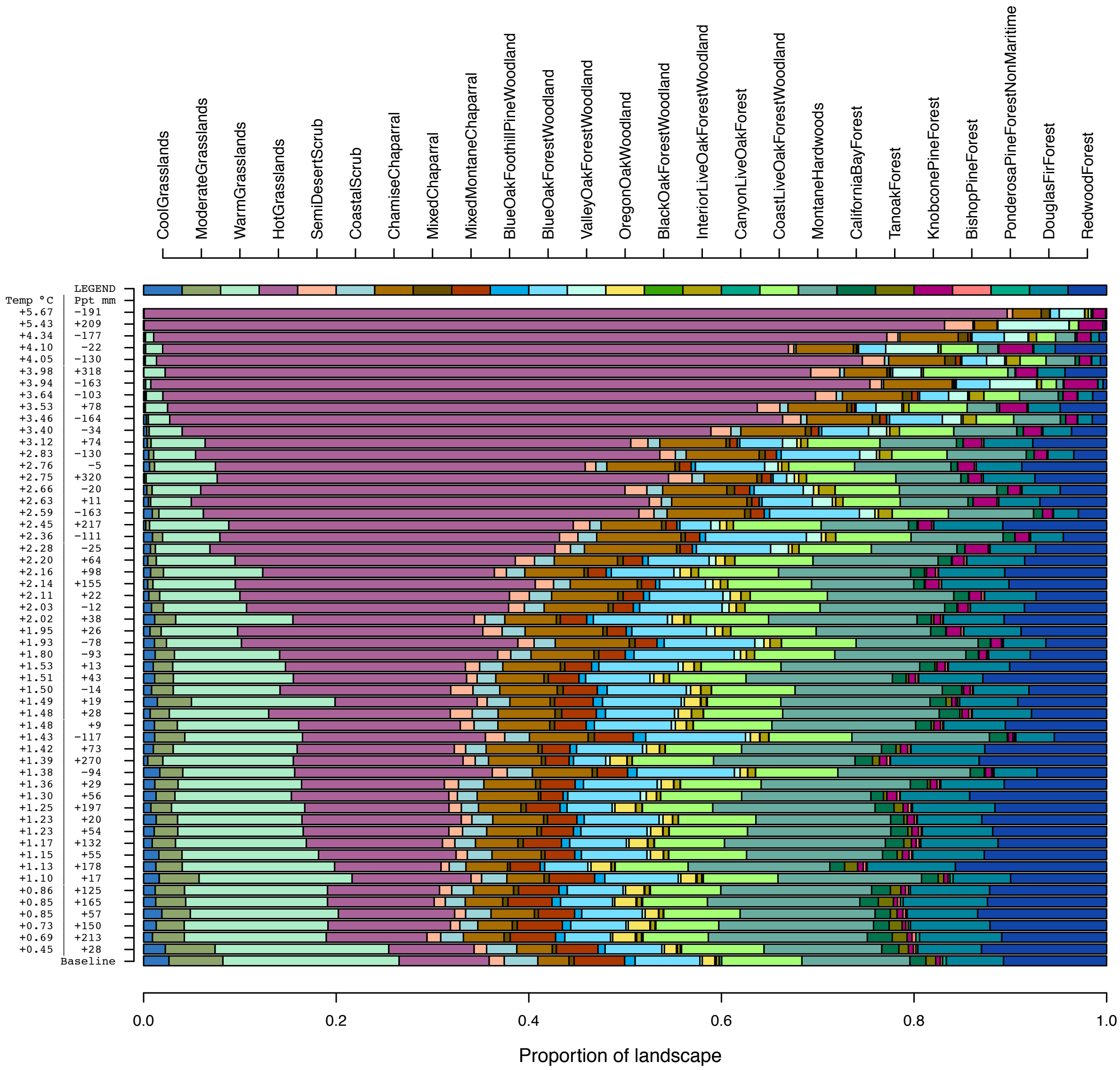

S5 Fig. b) ‘4G’ model, with grasslands splits into cool, moderate, warm and hot. Changes under a warmer and drier climate are dramatically different from the ‘0G’ and ‘1G’ models, as the hot grasslands expand to occupy almost the entire region. Expansion of grasslands seems unlikely without extensive anthropogenic intervention by grazing and management to prevent woody succession. More broadly, the models are in agreement to the extent that they all predict increased dominance of one or more of the various vegetation types that currently occupy hot, interior regions.
