# Supplementary material for: A Geographic Mosaic of Climate Change Impacts on Terrestrial Vegetation: Which Areas Are Most at Risk?
Source: PLoS One. 2015 Jun 26;10(6):e0130629. doi: 10.1371/journal.pone.0130629 (PMC4482696; doi:10.1371/journal.pone.0130629)
Supplement: S6 Fig — (PDF) [file pone.0130629.s006.pdf]

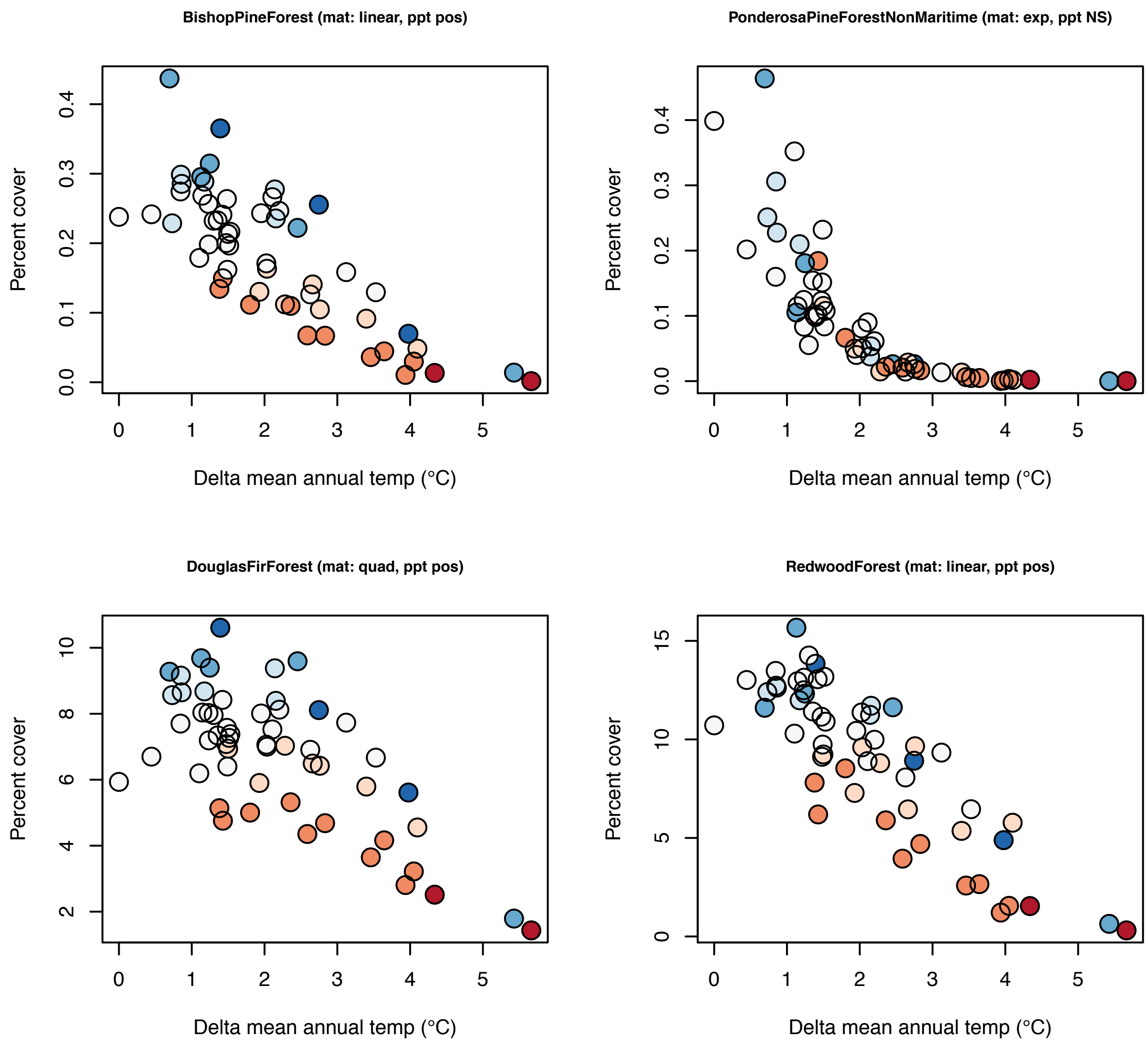


S6 Fig. Changes in relative abundance of each of the 22 vegetation types, plotted relative to mean annual temperature (MAT) of each of the climate scenarios. Colors indicate change in precipitation (see legend in Fig. 4). The title for each panel indicates the vegetation type, shown in the same order as Figure 3, and the shape and sign of the regressions relative to MAT and PPT. All regressions with respect to MAT were significant except Mixed Chaparral (see Table S4). Abbreviations: quad = quadratic; exp = exponential; neg = negative; pos = positive; NS = non-significant relationships.
