# Supplementary material for: A Geographic Mosaic of Climate Change Impacts on Terrestrial Vegetation: Which Areas Are Most at Risk?
Source: PLoS One. 2015 Jun 26;10(6):e0130629. doi: 10.1371/journal.pone.0130629 (PMC4482696; doi:10.1371/journal.pone.0130629)
Supplement: S7 Fig — (PDF) [file pone.0130629.s007.pdf]

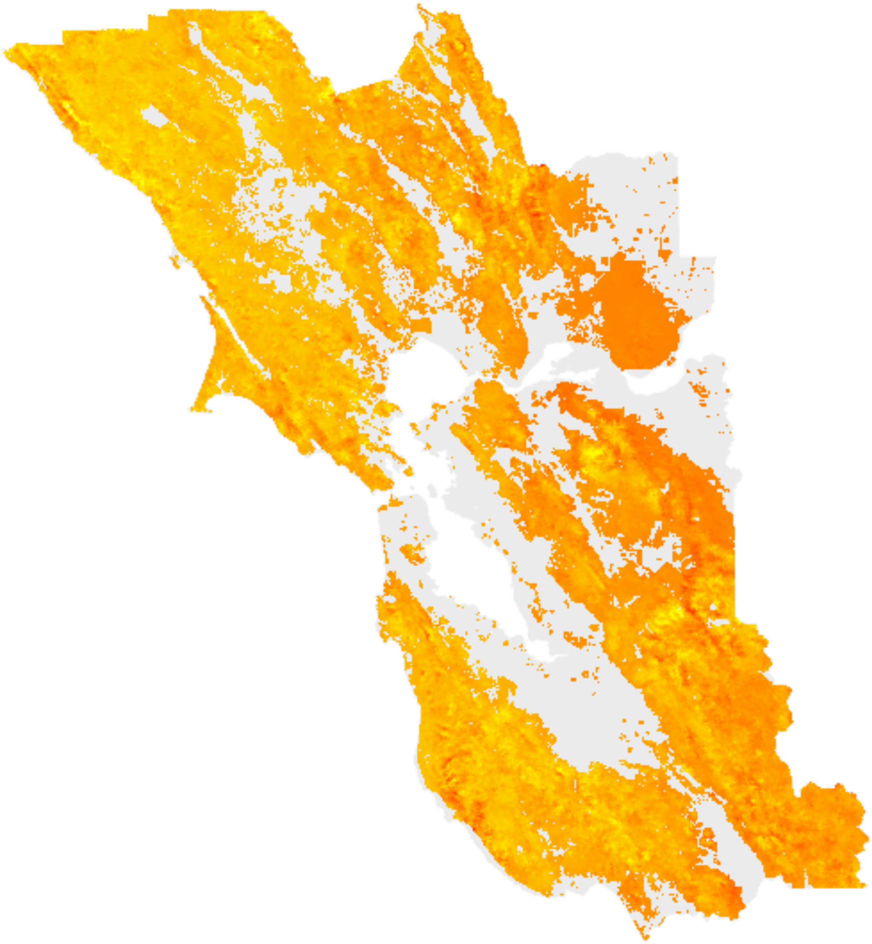

S7 Fig. Geographic variation in the rate of climate change, based on a regression of local changes in MAT vs. the regional means. Higher values (red) indicate more rapid change.
