# Supplementary material for: A Geographic Mosaic of Climate Change Impacts on Terrestrial Vegetation: Which Areas Are Most at Risk?
Source: PLoS One. 2015 Jun 26;10(6):e0130629. doi: 10.1371/journal.pone.0130629 (PMC4482696; doi:10.1371/journal.pone.0130629)
Supplement: S8 Fig — (PDF) [file pone.0130629.s008.pdf]

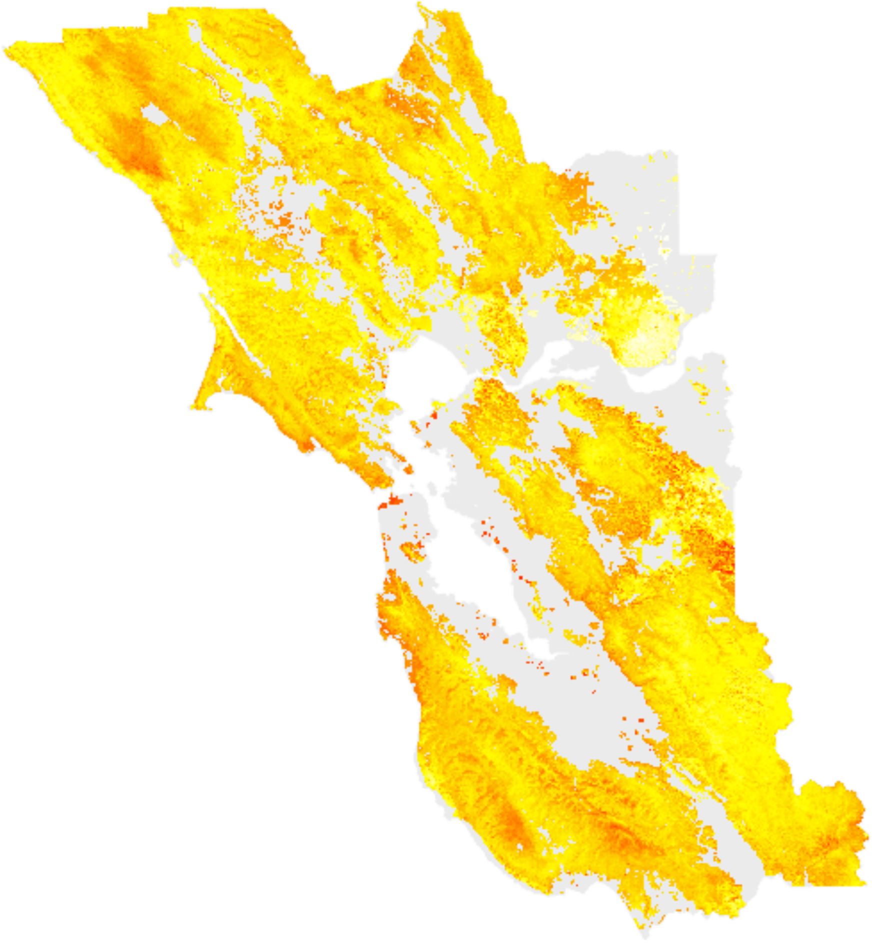

S8 Fig. Geographic variation in the projected impact of climate change on vegetation, based on the product of sensitivity of vegetation to local climate change (Fig. 8) and the magnitude of change (Fig. S6). Higher values in red.
