# Supplementary material for: A Geographic Mosaic of Climate Change Impacts on Terrestrial Vegetation: Which Areas Are Most at Risk?
Source: PLoS One. 2015 Jun 26;10(6):e0130629. doi: 10.1371/journal.pone.0130629 (PMC4482696; doi:10.1371/journal.pone.0130629)
Supplement: S9 Fig — (PDF) [file pone.0130629.s009.pdf]

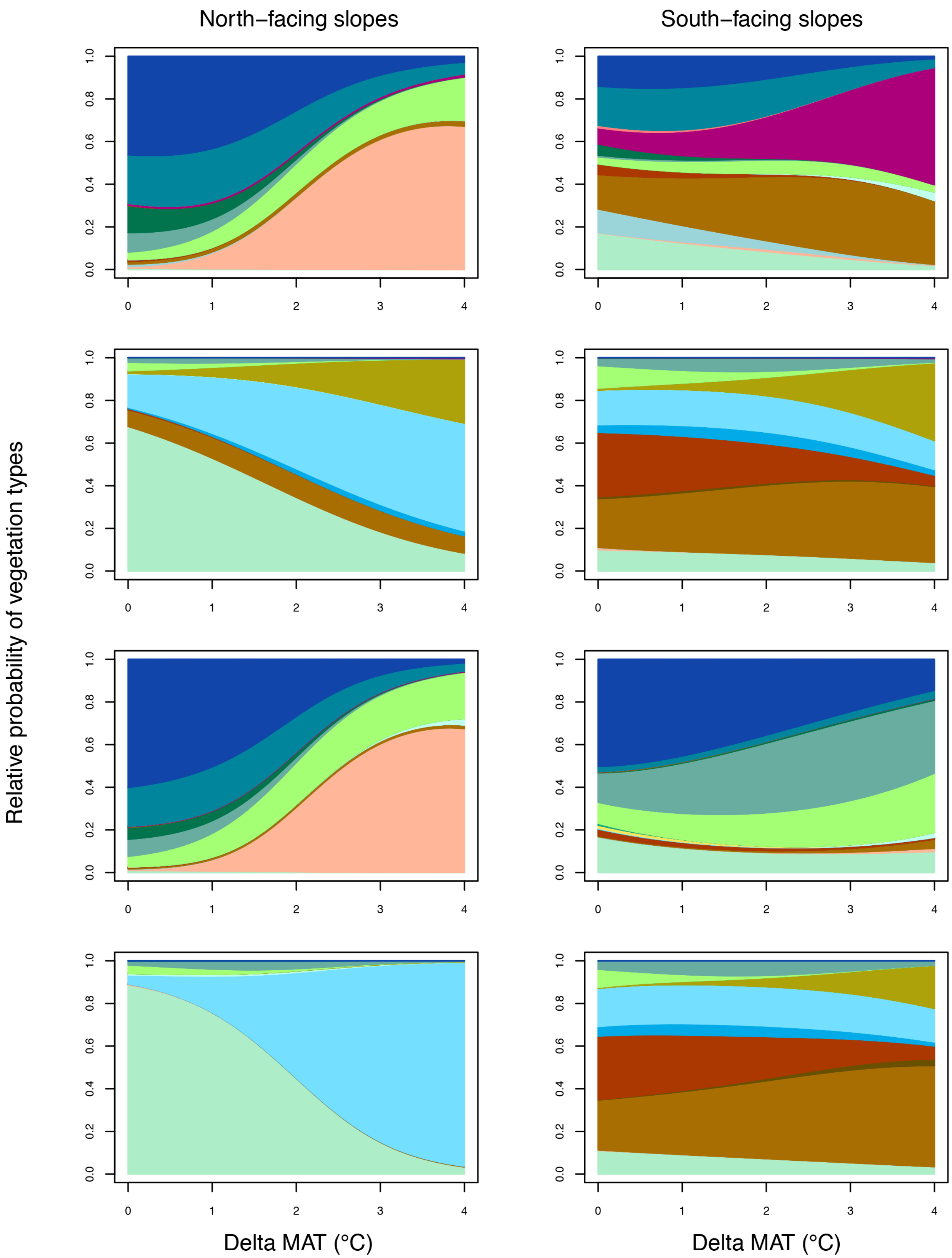

North–facing slopes
South–facing slopes
Relative probability of vegetation types
Delta MAT (°C)
Delta MAT (°C)

S9 Fig. Illustrations of projected sensitivity of north-facing vs. south-facing slopes in relation to constructed future climates with JJA, DJF and CWD increasing in proportion to MAT (see Fig. 2). PPT was kept constant in these scenarios, as were soil depth, insolation, and wind, at the observed values for each pixel. Each row is a pair selected in fairly close geographic proximity. Colors correspond to vegetation types, as shown in Fig. 3.
