# Supplementary material for: A Geographic Mosaic of Climate Change Impacts on Terrestrial Vegetation: Which Areas Are Most at Risk?
Source: PLoS One. 2015 Jun 26;10(6):e0130629. doi: 10.1371/journal.pone.0130629 (PMC4482696; doi:10.1371/journal.pone.0130629)
Supplement: S1 Table — (PDF) [file pone.0130629.s010.pdf]

S10 Table. Vegetation types in alphabetical order with scientific names of dominant taxa. 1G and 4G numbers identify vegetation types with 1 or 4 grassland types (see Methods, and types listed in supplemental data on Dryad). 1G numbers correspond to symbols in Fig. S2. Scientific names follow Baldwin et al. 2012.

**Number (‘1G’)**	**Number (‘4G’)**	**Physiognomic types (5)**	**Vegetation types**	**Scientific names of dominant taxa**
1	1	Conifer	Bishop Pine Forest	*Pinus muricata*
2	2	Dec. Woodland	Black Oak Forest/Woodland	*Quercus kelloggii*
3	3	Dec. Woodland	Blue Oak Foothill Pine Woodland (M)	*Quercus douglasii / Pinus sabiniana*
4	4	Dec. Woodland	Blue Oak Forest Woodland	*Quercus douglasii*
5	5	Evergreen Woodland	California Bay Forest	*Umbellularia californica*
6	6	Evergreen Woodland	Canyon Live Oak Forest	*Quercus chrysopelis*
7	7	Shrubland	Chamise Chaparral	*Adenostoma fasciculatum*
8	8	Shrubland	Coastal Scrub	Various species
9	9	Evergreen Woodland	Coast Live Oak Forest/Woodland	*Quercus agrifolia*
NA	10	Grassland	Cool Grassland	Various species
10	11	Conifer	Douglas Fir Forest	*Pseudostuga menziesii*
11	NA	Grassland	Grassland	Various species
NA	12	Grassland	Hot Grassland	Various species
12	13	Evergreen Woodland	Interior Live Oak Forest/Woodland	*Quercus wislezini*
13	14	Conifer	Knobcone Pine Forest	*Pinus attenuata*
14	15	Shrubland	Mixed Chaparral	Various species
15	16	Shrubland	Mixed Montane Chaparral	Various species
NA	17	Grassland	Moderate Grassland	Various species
16	18	Evergreen Woodland	Montane Hardwoods (M)	Various species
17	19	Dec. Woodland	Oregon Oak Woodland	*Quercus garryana*
18	20	Conifer	Ponderosa Pine Forest (Non-Maritime)	*Pinus ponderosa*
19	21	Conifer	Redwood Forest	*Sequoiadendron*

				sempervirens
20	22	Shrubland	Semi-Desert Scrub	Various species
21	23	Evergreen Woodland	Tanoak Forest	*Notholithocarpus densiflorus*
22	24	Dec. Woodland	Valley Oak Forest Woodland	*Quercus lobata*
NA	25	Grassland	Warm Grassland	Various species
