# Supplementary material for: A Geographic Mosaic of Climate Change Impacts on Terrestrial Vegetation: Which Areas Are Most at Risk?
Source: PLoS One. 2015 Jun 26;10(6):e0130629. doi: 10.1371/journal.pone.0130629 (PMC4482696; doi:10.1371/journal.pone.0130629)
Supplement: S2 Table — (PDF) [file pone.0130629.s011.pdf]

S11 Table. Mean and range of environmental parameters for historical baseline period, sampled over 1 million points used for model projection.

		Baseline (1951-1980)			Change in mean over 54 future scenarios	
Variable	units	Mean	Min	Max	Min	Max
Elevation	m	317	-3	1359	-	-
March insolation	Wh m^{-2}	3475	437	4783	-	-
Soil depth	m	0.904	0.10	4	-	-
Wind speed	m s^{-1}	4.18	1.25	9.06	-	-
DJF (winter min temp)	°C	4.18	0.993	7.48	+0.47	+5.86
JJA (summer max temp)	°C	27.9	16.2	34.5	+0.14	+6.61
Climatic water deficit	mm	786	273	1046	-31.9	+184
Precipitation	mm	847	280	1989	-172	+292
