# Supplementary material for: A Geographic Mosaic of Climate Change Impacts on Terrestrial Vegetation: Which Areas Are Most at Risk?
Source: PLoS One. 2015 Jun 26;10(6):e0130629. doi: 10.1371/journal.pone.0130629 (PMC4482696; doi:10.1371/journal.pone.0130629)
Supplement: S3 Table — (PDF) [file pone.0130629.s012.pdf]

S12 Table. Mean of climate parameters across all historical and future scenarios, averaged over 1 million sample points used in model. Scenarios are sorted by MAT.

N	Model	Forcing	Time Period	DJF (°C)	JJA (°C)	CWD (mm)	PPT (mm)	MAT (°C)	delta MAT (°C)
1	HST	HST	1951_1980	4.18	27.92	786	847	14.39	0.00
2	HST	HST	1981_2010	4.64	28.00	798	875	14.84	0.45
26	GISS	rcp26	2070_2099	4.91	28.47	759	1060	15.08	0.69
25	GISS	rcp26	2040_2069	4.64	29.08	798	997	15.12	0.73
21	GISS_aom	A1B	2010_2039	4.99	28.62	812	904	15.23	0.85
24	GISS	rcp26	2010_2039	4.77	28.71	785	1012	15.24	0.85
48	mri_cgcm3	rcp26	2010_2039	4.93	28.79	793	972	15.25	0.86
51	PCM	A2	2010_2039	4.63	28.81	800	864	15.49	1.10
54	PCM	B1	2010_2039	5.12	28.66	812	1025	15.52	1.13
9	CSIRO_mk35	A1B	2010_2039	5.25	28.86	818	902	15.53	1.15
49	mri_cgcm3	rcp26	2040_2069	5.10	29.20	796	979	15.56	1.17
3	CCSM4	rcp85	2010_2039	5.34	29.07	813	901	15.62	1.23
42	MIROC5	rcp26	2010_2039	5.30	29.25	832	867	15.62	1.23
50	mri_cgcm3	rcp26	2070_2099	5.25	29.24	793	1044	15.64	1.25
18	GFDL	B1	2010_2039	5.62	29.03	839	903	15.69	1.30
15	GFDL	A2	2010_2039	5.32	29.13	812	877	15.75	1.36
39	Miroc_Medres	A2	2010_2039	5.37	29.52	837	754	15.77	1.38
6	CNRM	rcp85	2010_2039	5.76	29.14	799	1117	15.78	1.39
45	mpi_esm_lr	rcp45	2010_2039	5.41	29.21	822	920	15.81	1.42
33	MIROC_esm	rcp60	2010_2039	5.17	29.57	820	731	15.82	1.43
12	Fgoals_g2	rcp85	2010_2039	5.32	29.32	821	856	15.86	1.48
30	MIROC_esm	rcp45	2010_2039	5.53	29.66	799	875	15.87	1.48
55	PCM	B1	2040_2069	4.82	29.17	812	867	15.88	1.49
36	MIROC_esm	rcp85	2010_2039	5.49	29.48	807	833	15.89	1.50
27	IPSL_CM5A_LR	rcp85	2010_2039	5.34	29.52	835	890	15.90	1.51
43	MIROC5	rcp26	2040_2069	5.55	29.38	824	860	15.92	1.53
44	MIROC5	rcp26	2070_2099	5.50	29.85	848	755	16.19	1.80
22	GISS_aom	A1B	2040_2069	5.95	30.13	843	769	16.32	1.93
19	GFDL	B1	2040_2069	6.21	29.54	828	873	16.34	1.95
52	PCM	A2	2040_2069	5.46	29.56	832	885	16.41	2.02
4	CCSM4	rcp85	2040_2069	5.89	30.08	844	835	16.42	2.03
46	mpi_esm_lr	rcp45	2040_2069	6.05	29.97	824	869	16.50	2.11
10	CSIRO_mk35	A1B	2040_2069	6.34	29.92	828	1002	16.53	2.14
56	PCM	B1	2070_2099	5.95	29.68	827	945	16.54	2.16
47	mpi_esm_lr	rcp45	2070_2099	6.10	30.16	827	911	16.59	2.20
16	GFDL	A2	2040_2069	6.58	30.22	868	822	16.67	2.28
20	GFDL	B1	2070_2099	6.38	30.10	856	736	16.75	2.36
7	CNRM	rcp85	2040_2069	6.53	30.32	835	1064	16.84	2.45
40	Miroc_Medres	A2	2040_2069	6.43	30.90	870	685	16.98	2.59
23	GISS_aom	A1B	2070_2099	6.73	31.02	877	858	17.02	2.63
34	MIROC_esm	rcp60	2040_2069	6.47	31.09	844	827	17.05	2.66
11	CSIRO_mk35	A1B	2070_2099	6.85	30.68	815	1168	17.14	2.75
13	Fgoals_g2	rcp85	2040_2069	6.24	30.92	880	842	17.15	2.76
31	MIROC_esm	rcp45	2040_2069	6.53	31.23	878	718	17.22	2.83
53	PCM	A2	2070_2099	6.71	30.71	862	921	17.51	3.12
28	IPSL_CM5A_LR	rcp85	2040_2069	6.86	31.39	873	813	17.79	3.40
37	MIROC_esm	rcp85	2040_2069	6.99	32.11	896	684	17.85	3.46
5	CCSM4	rcp85	2070_2099	7.44	31.50	877	925	17.92	3.53
32	MIROC_esm	rcp45	2070_2099	7.23	32.40	883	744	18.03	3.64
17	GFDL	A2	2070_2099	8.12	31.76	930	684	18.33	3.94
8	CNRM	rcp85	2070_2099	8.18	31.91	870	1165	18.37	3.98
35	MIROC_esm	rcp60	2070_2099	7.42	32.89	893	717	18.44	4.05
14	Fgoals_g2	rcp85	2070_2099	7.45	32.45	943	826	18.49	4.10
41	Miroc_Medres	A2	2070_2099	7.50	33.29	936	670	18.73	4.34
29	IPSL_CM5A_LR	rcp85	2070_2099	9.98	33.03	906	1056	19.82	5.43
38	MIROC_esm	rcp85	2070_2099	8.74	34.51	962	657	20.05	5.67
