# Supplementary material for: A Geographic Mosaic of Climate Change Impacts on Terrestrial Vegetation: Which Areas Are Most at Risk?
Source: PLoS One. 2015 Jun 26;10(6):e0130629. doi: 10.1371/journal.pone.0130629 (PMC4482696; doi:10.1371/journal.pone.0130629)
Supplement: S4 Table — (PDF) [file pone.0130629.s013.pdf]

S13 Table. Nine pairs of vegetation types with correlations in predicted suitability under historical baseline conditions ≥ 0.5, indicating that suitable conditions occur in similar locations on the landscape.

Type 1	Type 2	Correlation
Blue oak / Foothill pine woodland	Blue oak forest and woodland	0.69
Blue oak / Foothill pine woodland	Interior live oak woodland	0.59
Blue oak forest and woodland	Interior live oak woodland	0.52
Chamise chaparral	Mixed montane chaparral	0.53
Coast live oak woodland	Interior desert shrubland	0.51
Douglas fir forest	Oregon oak woodland	0.52
Douglas fir forest	Tanoak forest	0.52
Montane hardwoods	Oregon oak woodland	0.57
