# Supplementary material for: A Geographic Mosaic of Climate Change Impacts on Terrestrial Vegetation: Which Areas Are Most at Risk?
Source: PLoS One. 2015 Jun 26;10(6):e0130629. doi: 10.1371/journal.pone.0130629 (PMC4482696; doi:10.1371/journal.pone.0130629)
Supplement: S5 Table — (PDF) [file pone.0130629.s014.pdf]

S14 Table. Results of multiple regression of change in area for each vegetation type as a function of MAT and PPT. If the MAT^2 coefficient is non-zero, quadratic regression was used. If 'log' is 1, the cover values were log-transformed and an exponential model was used.

	Model Coefficients				Model p-values				
VegType	Intercept	MAT^2	MAT	PPT	MAT^2	MAT	PPT	Adj R2	log
Grassland	134.0	0.000	-5.57E+00	-2.02E-02	NA	0.000	0.000	0.909	0
Semi-Desert Scrub	-9.2	0.000	5.73E-01	6.86E-04	NA	0.000	0.090	0.909	1
Coastal Scrub	32.7	0.070	-3.00E+00	-7.78E-04	0.001	0.000	0.019	0.876	0
Chamise Chaparral	86.5	0.515	-1.29E+01	-6.55E-03	0.000	0.001	0.000	0.946	0
Mixed Chaparral	0.8	0.000	1.44E-02	-6.21E-04	NA	0.416	0.001	0.208	0
Mixed Montane Chaparral	10.9	0.000	-5.52E-01	-1.05E-03	NA	0.000	0.000	0.208	1
Blue Oak-Foothill Pine Woodland	5.1	0.000	-1.36E-01	-2.18E-03	NA	0.000	0.000	0.660	0
Blue Oak Forest/Woodland	0.6	0.000	2.09E+00	-2.83E-02	NA	0.000	0.000	0.894	0
Valley Oak Forest/Woodland	-11.8	0.000	6.34E-01	5.89E-04	NA	0.000	0.155	0.894	1
Oregon Oak Woodland	4.5	0.000	-4.00E-01	3.91E-03	NA	0.000	0.000	0.751	0
Black Oak Forest/Woodland	26.0	0.000	-1.81E+00	-5.97E-04	NA	0.000	0.429	0.751	1
Interior Live Oak Forest/Woodland	-7.3	0.000	5.81E-01	-2.48E-03	NA	0.000	0.000	0.751	1
Canyon Live Oak Forest	27.7	0.000	-2.10E+00	2.41E-03	NA	0.000	0.003	0.751	1
Coast Live Oak Forest/Woodland	-257.9	-0.807	2.86E+01	1.80E-02	0.000	0.000	0.000	0.563	0
Montane Hardwoods	-206.1	-0.873	2.75E+01	6.54E-03	0.000	0.000	0.002	0.799	0
California Bay Forest	6.5	0.000	-3.29E-01	-7.53E-04	NA	0.000	0.039	0.751	1
Tanoak Forest	28.1	0.000	-2.03E+00	3.34E-03	NA	0.000	0.002	0.751	1
Knobcone Pine Forest	-9.7	0.000	5.57E-01	3.86E-04	NA	0.000	0.215	0.751	1
Bishop Pine Forest	0.7	0.000	-5.35E-02	3.61E-04	NA	0.000	0.000	0.814	0
Ponderosa Pine Forest-Non-Maritime	25.3	0.000	-1.67E+00	-1.14E-03	NA	0.000	0.147	0.814	1
Douglas Fir Forest	-78.1	-0.316	9.87E+00	9.62E-03	0.000	0.000	0.000	0.856	0
Redwood Forest	39.0	0.000	-2.41E+00	1.12E-02	NA	0.000	0.000	0.835	0
